# Battery-free, tuning circuit–inspired wireless sensor systems for detection of multiple biomarkers in bodily fluids

**DOI:** 10.1126/sciadv.abo7049

**Published:** 2022-07-06

**Authors:** Tzu-Li Liu, Yan Dong, Shulin Chen, Jie Zhou, Zhenqiang Ma, Jinghua Li

**Affiliations:** 1 Department of Materials Science and Engineering, The Ohio State University, Columbus, OH 43220, USA.; 2 Department of Electrical and Computer Engineering, University of Wisconsin-Madison, Madison, WI 53706, USA.; 3 Department of Materials Science and Engineering, University of Wisconsin-Madison, Madison, WI 53706, USA.; 4 Department of Engineering Physics, University of Wisconsin-Madison, Madison, WI 53706, USA.; 5 Chronic Brain Injury Program, The Ohio State University, Columbus, OH 43220, USA.

## Abstract

Tracking the concentration of biomarkers in biofluids can provide crucial information about health status. However, the complexity and nonideal form factors of conventional digital wireless schemes impose challenges in realizing biointegrated, lightweight, and miniaturized sensors. Inspired by the working principle of tuning circuits in radio frequency electronics, this study reports a class of battery-free wireless biochemical sensors: In a resonance circuit, the coupling between a sensing interface and an inductor-capacitor oscillator through a pair of varactor diodes converts a change in electric potential into a modulation in capacitance, resulting in a quantifiable shift of the resonance circuit. Proper design of sensing interfaces with biorecognition elements enables the detection of various biomarkers, including ions, neurotransmitters, and metabolites. Demonstrations of “smart accessories” and miniaturized probes suggest the broad utility of this circuit model. The design concepts and sensing strategies provide a realistic pathway to building biointegrated electronics for wireless biochemical sensing.

## Teaser

This study reports a class of wireless, lightweight, and multifunctional chemical sensors for detection of biomarkers.

## INTRODUCTION

The grand societal challenges regarding the ever-increasing demand for and cost of health care have motivated continued research efforts to develop personalized, precise, and preventive medicine. To this end, biointegrated sensing technologies play important roles in monitoring patients’ vital signs and detecting important biological anomalies. While commercial wearable and implantable sensors have evolved tremendously in recent years in monitoring various biophysical signals, the sensitive, selective, and wireless detection of chemical biomarkers in bodily fluids is worth further study for obtaining insight into physiological and pathological pathways at molecular levels. Bodily fluids contain many different types of biomarkers that are important for health monitoring and disease diagnosis (*1*–*4*). For example, sweat is a highly attractive candidate due to its easy accessibility and the rich biochemical information it carries. The level of multiple electrolytes and metabolites can serve as biomarkers for the monitoring of physical and emotional health status (*5*–*7*). Similarly, the detection of neurotransmitters and hormones is one of the most promising techniques in understanding brain functions (*8*–*10*). Consequently, developing wireless and biointegrated sensing technologies could potentially shift the paradigm of health care from a slow, centralized, and generic mode to a rapid, low-cost, and personalized one.

In recent years, studies on developing novel biochemical sensors and/or integrated systems with supporting wireless modules have received considerable attention and are progressing rapidly (*8*, *11*–*21*). The great success and breakthroughs of pioneering works in this field have set the stage for next-generation health care. Nevertheless, the following issues are worth further investigation: (i) Most currently available digital wireless schemes compatible with chemical sensing technologies require the use of commercial electronic components (e.g., operational amplifier and microcontrollers) for signal generation/transmission and batteries/energy-harvesting systems for power supply, with conventional rigid, printed circuit boards serving as mounting sites (*22*). The nonideal form factors, including the size, weight, and rigidity of the subsystems may constrain natural motions. The high level of complexity of the circuit layouts also imposes practical challenges in seamless integration with skin/other biotissues and precise interpretation of experimental results. In this context, passive resonance circuits are of great interest because of the simple, lightweight, battery-free, and miniaturizable structure suitable for serving as wireless wearable/implantable electronics (*23*). In these systems, target signals modulate a responsive element [i.e., inductance (*24*), capacitance (*25*–*27*), or resistance (*28*–*32*)] in the circuit, which then results in a change in the characteristics around the resonance peak (i.e., amplitude and/or frequency). Consequently, it is highly desirable to develop a general sensing strategy using resonance circuits for detection of various biomarkers. (ii) In addition to the wireless sensing capability, desired key performance metrics should include sensitivity, selectivity, and multifunctionality to support concurrent recording of multiple biochemical signals with a minimal level of cross-talk for establishing biometric signature profiles associated with health status. (iii) For applications as wearable/implantable electronics, how to minimize motion/strain/environment-induced changes in the electrical performance of the circuit requires further attention. Overall, addressing these issues requires joint efforts in the proper design of both biochemical interfaces and electronic transducers, as well as the coupling strategy between them.

To tackle these challenges, this study reports a class of biointegrated wireless chemical sensors inspired by the working principle of tuning circuits in radio frequency (RF) electronics, with major innovations described as follows: The system includes an inductor-capacitor (LC) resonance circuit consisting of a coil and a pair of varactor diodes electrically coupled with a functionalized sensing interface. The varactors convert a change in electric potential caused by surface biochemical events into a capacitance modulation, which can then be quantified by reading the shift in the resonance frequency (*f*_s_) of the LC circuit. The fundamental difficulty that this work addresses is how to design lightweight, simple, wireless, and biointegrated chemical sensors. State-of-the-art digital wireless schemes usually require integrated electronic components with nonideal form factors for signal generation/transmission (*22*). While passive sensors working based on resonant inductive coupling have merits due to the simple configuration and light weight, conventional designs usually have the responsive elements integrated within the LC circuit. As a result, it is difficult to eliminate artifacts caused by interactions between the electromagnetic coupling unit and target biotissues/biofluids. The innovation of this work is the successful demonstration of a modularized LC circuit model where a pair of varactors separates the device into a coupling unit and a sensing interface connected by extended wires. The advantage of the system over existing systems in the literature includes battery-free operation, light weight, and improved stability of the coupling unit for passive sensing systems. Therefore, although stretchable electronics design, varactor diode–based glucose sensors (*33*), wearable electronics, multiple sensing interfaces, and biosensors for various analytes (e.g., ions and neurotransmitters) are previously reported concepts, the unprecedented combination of them for bridging the knowledge gap represents the major innovation and uniqueness of this study. The design concepts here provide a promising route to building versatile, lightweight, and wireless sensing platforms compatible with multiple application scenarios. By properly designing the sensing interfaces using corresponding biorecognition elements [e.g., ion-selective membranes (ISMs), aptamers, and enzymes] to induce a potential change proportional to the concentration of biomarkers, this sensing strategy can detect a variety of analytes, such as ions, neurotransmitters, and metabolites. As a result, this system can serve as a general and versatile biochemical sensing platform. Demonstration of a “smart necklace” following this device concept integrated with functional “pendant,” “clasp,” and “chain” illustrates real-time monitoring of glucose in sweat during field testing. The study also shows that further miniaturizing the circuit model to form probe-type sensors is possible for potential applications as bioimplants in the future. Overall, by wirelessly measuring biomarker concentrations associated with the response of the body to the environment, stress, and diseases, this simple and versatile sensor system can find broad applications in biomedical research and clinical practices.

## RESULTS

### Design and working principle of the LC circuit–based wireless biochemical sensors

**Fig. 1.**
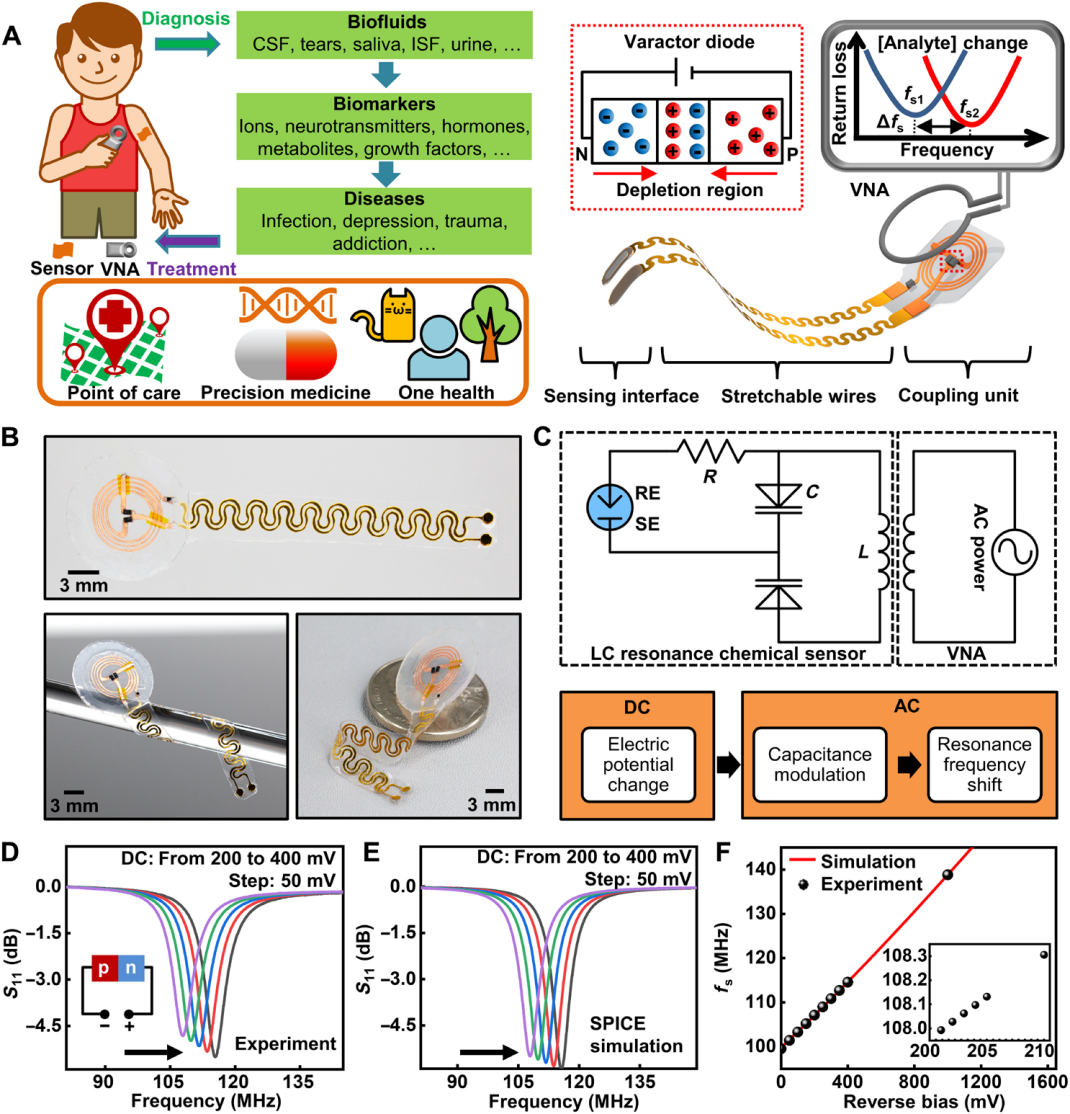
Design and working principle of the tuning circuit–inspired wireless biochemical sensors. (**A**) Schematic illustration of the stretchable, battery-free sensor and envisioned applications of the sensor system in detecting various biomarkers in bodily fluids. ISF, Interstitial fluid. (**B**) Photographs of a wireless sensor. (**C**) Equivalent circuit and flowchart for the signal conversion and transmission process of the sensor system. (**D** and **E**) Measured and simulated shift of resonance curves of a sensor with a DC reverse bias (from 200 to 400 mV; step: 50 mV). (**F**) Measured and simulated results of *f*_s_ as a function of the reverse bias (inset: zoom-in view).

Figure 1A shows the schematic illustration, working principle, and envisioned applications of the wireless LC circuit–based biochemical sensors prepared by a simple cut-and-paste method (fig. S1) (*24*, *34*, *35*). The device consists of two key functional parts: a stretchable biochemical sensing interface (DC part) to be in contact with target biofluids and a loop antenna as the coupling unit (AC part) for wireless signal transmission. Photographs and the equivalent circuit diagram of a flexible device appear in Fig. 1 (B and C), respectively. Stretchable wires connecting the DC and AC parts can effectively distribute mechanical strains and protect functional components from deformations. The working principle of tuning circuits in RF electronics inspires the design based on the resonance between an inductor and a pair of varactor diodes (*36*): The interaction between analytes and the corresponding biorecognition elements creates a potential difference across the two electrodes in the DC part that scales with the concentration of target biochemical species. The potential serves as the reverse bias for the two series varactor diodes. The varactors then convert the change in potential into a modulation in the capacitance of the diodes by modifying the thickness of the depletion layer in the p-n junctions. According to the datasheet of the varactor diode used in this study (SMV1249), the inductance is a constant value (*L*_s_ = 10 fH), which is related to metal contacts, while the capacitance varies as a function of the input DC bias. Therefore, the change in the thickness of the depletion layer only affects the capacitance and not the inductance. The head-to-head (or back-to-back) varactor configuration eliminates the DC path through the inductor and prevents AC signals from modulating the DC voltage by canceling out RF voltage–induced capacitance variation. When needed, replacing one varactor with a capacitor can also form functional devices. The modulation in capacitance induced by the bias voltage leads to a shift in *f*_s_ of the resonance circuit according to the following equation

$$f_{s}=\frac{1}{2\pi \sqrt{\mathrm{LC}}}$$ (1)

where *L* and *C* are the inductance and capacitance of the resonance circuit, respectively. For quantification, coupling the inductor to an alternating electromagnetic field through a readout coil enables the recording of the input return loss (*S*_11_) using a vector network analyzer (VNA). Characterizing the signal reflectance magnitude (*S*_11_ in decibels) yields a dip around *f*_s_, and fitting the curve estimates the value of *f*_s_.

This study uses commercial silicon hyperabrupt junction varactor diodes (SMV1249, Skyworks Solutions, Inc.; 1.0 mm by 0.6 mm by 0.46 mm) due to their high voltage sensitivity (i.e., variation of *C* with reverse bias) (*37*). Applying a reverse bias to the diodes allows for the evaluation of the electrical performance of the LC circuit in transducing DC signals. Figure 1 (D and E) shows measurement and simulation [performed using the Simulation Program with Integrated Circuit Emphasis (SPICE)] results obtained using the loop antenna shown in Fig. 1B, with a DC reverse bias ranging from 200 to 400 mV (step: 50 mV). The electromagnetic coupling unit has a *f*_s_ of ~110 MHz and a *Q* factor of ~15. With an increasing reverse bias, the width of the depletion layer increases. This leads to a decrease in the capacitance, which then results in an increasing *f*_s_ according to Eq. 1. The calibration function *f*_s_ (V) is near-linear when the reverse bias is small and in the range of physiologically meaningful signals caused by surface chemical events (typically <200 mV) (Fig. 1F, note S1, and fig. S2) (*38*). The system can clearly distinguish a difference in the input voltage of as low as 1 mV (Fig. 1F, inset), indicative of the capability of the system in detecting minute changes in biochemical signals. Beyond demonstrations with commercial varactor diodes in this study, developing low-cost biosensors in large scale demands simple and easily accessible fabrication schemes. The formation of hyperabrupt junctions in commercial varactor diodes, however, relies on well-calibrated and programmed molecular beam epitaxy techniques. This process requires delicate control and facilities, which most research laboratories have limited access to. On the other hand, preparing simple abrupt p-n junction diodes in an academic setup is much easier (*37*). A demonstration using a pair of SiGe/Si p-n diodes prepared in academic cleanroom appears in fig. S3 and note S2. The 1/*C*^2^-*V* plot suggests a linear relationship under reverse bias, which is consistent with the theoretical model (*37*). *f*_s_ also shows a monotonic change under forward bias as a result of modulation in diffusion capacitance. The demonstration with SiGe/Si p-n diodes contributes to this study by showing that these simple p-n junction diodes can also work for this circuit model and can fulfill the system requirements. By decoupling the dependence of the resonance circuit model based on commercial varactor diodes with the simplest p-n junctions, this result provides a realistic route toward preparing a lightweight and miniaturized biosensor system from the ground up using the easiest accessible Si-based technologies. Future efforts in developing thin-film diodes can further expand the utility of this circuit model as miniaturized, lightweight, and flexible sensor systems.

For applications of bioimplants, the wireless operation will generate electromagnetic field passing through biotissues. Calculation of the specific absorption rate (SAR) under the maximum output power of the VNA (−9 dBm) evaluates the electromagnetic safety of the sensor system (vertical distance between the tissue and the readout coil: 0.5 mm) (fig. S4A). The simulated SAR value (25 × 10^−6^ W/kg) is well below that deemed as a significant risk (4 W/kg per 15 min, averaged over whole body) according to the U.S. Food and Drug Administration regulation (*39*). In addition, it is also negligible when compared to the limit for public exposure to RF energy from wireless devices according to the Federal Communication Commission (1.6 W/kg) (*40*), implying the compliance of the sensor system with safety standards. Simulating and measuring heat generation during operation provide information about the thermal safety of the sensor system to biotissues. Simulation results show that the local temperature change is less than 1.68 × 10^−6^ K (fig. S4, B and C). Figure S4D shows the infrared thermal camera images of a sensor deployed on human before and after continuous operation for 30 min. The results show a minimal heating effect on the skin, suggesting a high level of thermal safety of the wireless sensing platform.

### Working range and mechanical properties of the wireless sensors

**Fig. 2.**
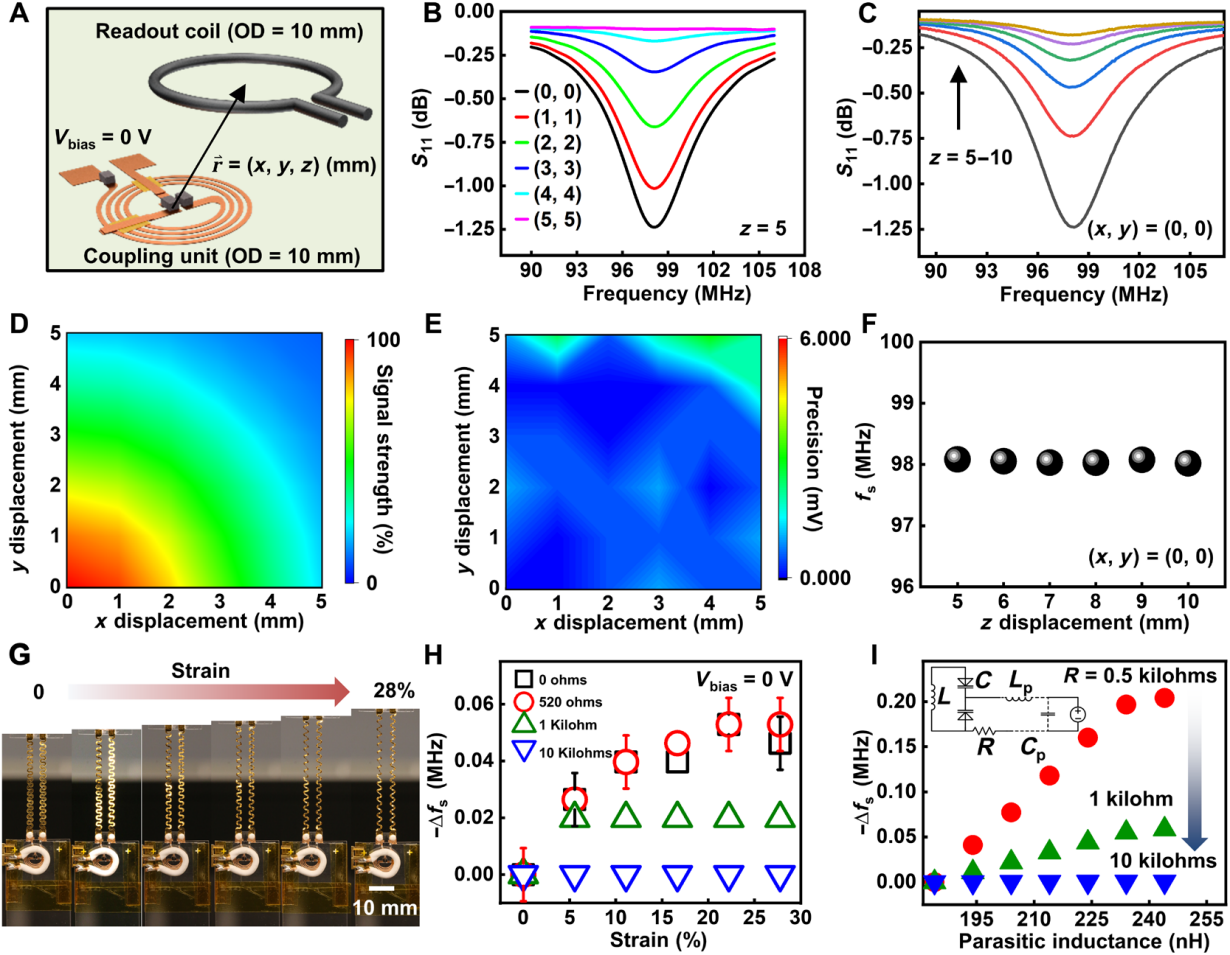
Working range and mechanical properties of the biochemical sensors. (**A**) Schematic illustration of the relative position of the sensing platform and the readout coil. (**B** and **C**) Resonance curves with varied lateral (B) and vertical (C) distance between the electromagnetic coupling unit and the readout coil. (**D** and **E**) Signal strength (D) and measurement precision (E) as a function of the lateral distance from the origin (*z* = 5 mm). (**F**) *f*_s_ as a function of the vertical distance (*x* = *y* = 0). (**G**) Photographs of a stretchable sensor with a tensile strain (from 0 to 28%) applied to the serpentine wires. (**H**) Measured results of *f*_s_ as a function of the damping resistance and applied strain. (**I**) SPICE simulation results of *f*_s_ as a function of the parasitic inductance (*L*_p_) and damping resistance (inset: equivalent circuit used for simulation).

One unique feature of this circuit model is that the system works based on a frequency modulation mechanism for chemical sensing by converting changes in electric potential into modulation in capacitance using the varactors, in contrast to an amplitude modulation one, relying on tuning the *Q* factor of the circuit. Although the magnitude of the resonance curve depends on the orientation and distance between the electromagnetic coupling unit and the readout coil that affect the coupling coefficient (*28*, *41*), *f*_s_ represents an intrinsic property of the resonance circuit and is relatively independent of the variation. Thus, it can provide reliable wireless signal transmission despite a slight misalignment between the coupling unit and primary coil. Characterizing electrical performances upon systematically changing the relative position between the coupling unit and the readout coil using a three-axis stage defines the operating range of an antenna with an outer diameter (OD) = 10 mm (Fig. 2A). In this test, shorting the cathode and anode of the varactors minimizes the impact of environmental noises and static electricity. Figure 2 (B and C) shows the resonance curves of the system with varied (*x*, *y*) and (*z*), respectively. The complete set of raw data for the systematic study is in fig. S5. Figure 2D shows the mapping of signal strength related to the mutual inductance between the sensor and the readout coil. The results qualitatively agree with the near-field simulation, showing the strength of the magnetic field around the inductor (fig. S6). The mapping of random noise level (defined as “measurement precision”) in Fig. 2E suggests a minimal impact (i.e., noise < 3 mV) of the relative position change on the performance of the sensing platform within the displacement range [(*x*, *y*) from (0, 0) to (5, 5) mm, *z* = 5 mm]. The value of *f*_s_ also remains almost constant [(*x*, *y*) = (0, 0)], with a varying vertical distance between the sensor and the readout coil (Fig. 2F).

A challenge for building biointegrated sensors using resonance circuits is that motion/environment-induced noises may cause variations in parasitic capacitance/inductance, resulting in an undesired shift in *f*_s_. The use of varactors addresses this issue by dividing the system into the two key functional parts connected by extended wires: the electromagnetic coupling unit for wireless signal transmission that can be encapsulated to provide stable electrical performance and the stretchable sensing interface in direct contact with target fluids/tissues. Placing a damping resistor in series with the sensing interface isolates the bias circuitry from the coupling unit, thereby suppressing parasitic oscillation and minimizing the strain/environment-induced changes in electrical properties of the transmission line. The rationale is that the resistance should be high enough for isolating the DC part from the coupling unit without lowering the *Q* factor of the overall circuitry: Here, the resistor is in parallel with the series LC tank. Decreasing the resistance will lead to a stronger damping effect, resulting in a lower *Q* due to an enhanced level of energy dissipation, and vice versa. Figure 2G and fig. S7 show a device with serpentine wires subject to a tensile strain ranging from 0 to 28%. Corresponding changes in *f*_s_ as a function of the strain and the resistance appear in Fig. 2H. With a resistance of 10 kilohms, the variation in *f*_s_ falls below the resolution of the VNA (20 kHz) throughout the test. The following part of this study uses this value for sensor development. SPICE simulation shows qualitatively similar results (Fig. 2I). Here, *L* refers to the inductor coil in the coupling unit, whereas *L*_P_ and *C*_P_ refer to the equivalent parasitic inductance and capacitance of the transmission lines in the DC part, respectively, which vary during stretching/bending (parameters used for simulation: parasitic capacitance *C*_p_ = 0.1 pF, parasitic inductance *L*_p_ from ~1.8 to 2.4 nH, *V*_bias_ = 0 mV). Going beyond the strain of ~30% allows for realizing the full potential of these devices as wearable electronics. Results show that the device can remain functional with a stable *f*_s_ and no obvious deleterious effects in the presence of a larger degree of strain up to 50% before it fractures at a strain of ~53% (fig. S8, A and B). The results suggest that the device can withstand relevant levels of bending/stretching. Cyclic stretch tests evaluate the mechanical robustness and reliability of the system. Figure S8C shows the extracted resonance frequency of a test wireless sensor before and after 0 to 8000 stretch cycles with an applied tensile strain of 30%. The results indicate that the system retains a stable electrical performance during the tests, providing the necessary flexibility and stretchability as biointegrated electronics.

### Interface design and sensing performance in response to various biomarkers

**Fig. 3.**
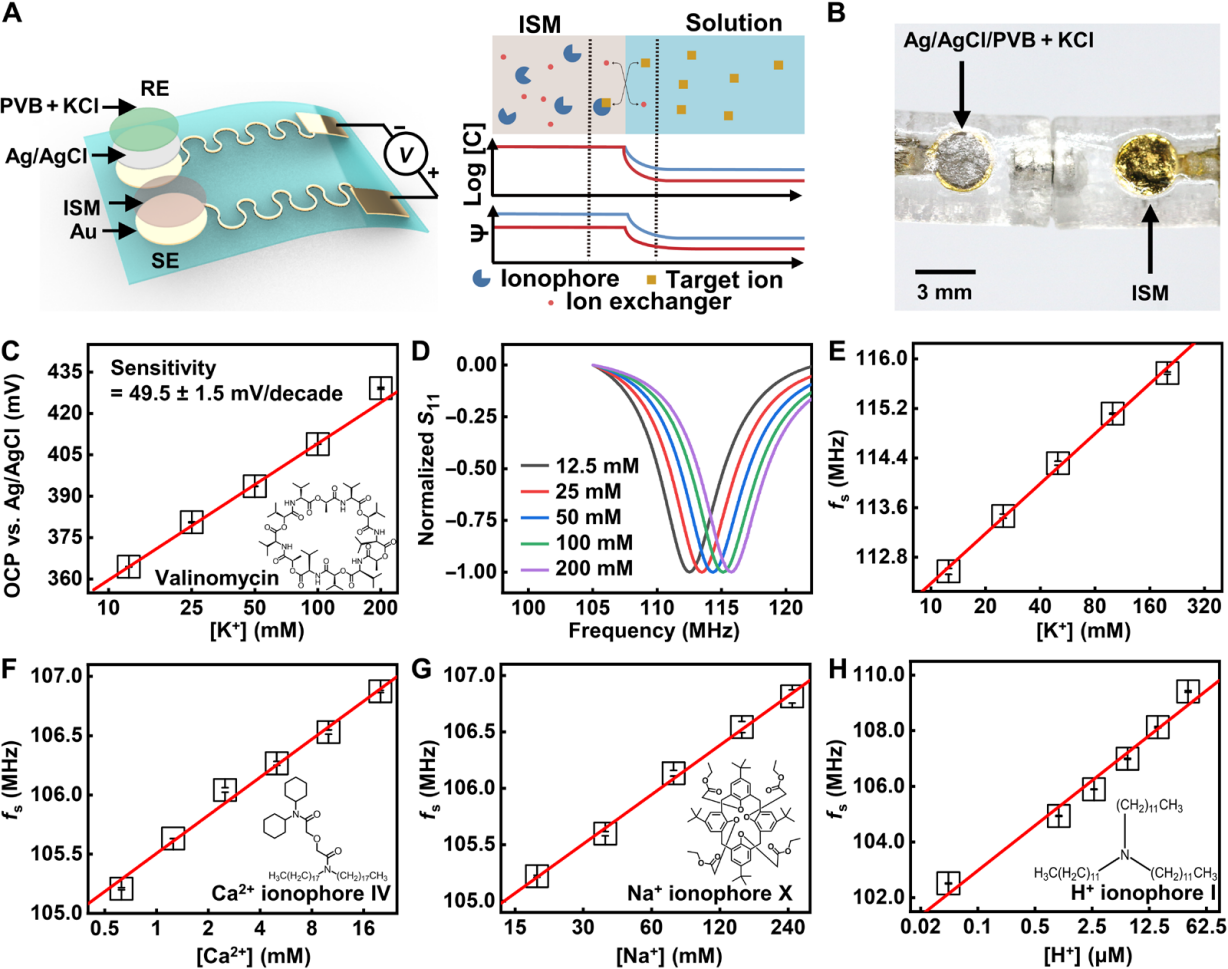
Sensing performance of ISM-functionalized wireless potentiometric sensors. (**A**) Schematic illustrations of the DC part of ISM-functionalized sensors and the interaction between ISM and ions at the solution-sensor interface. (**B**) Photograph of a pair of thin-film electrodes as SE and RE for potentiometric sensing. (**C**) OCP (versus a Ag/AgCl RE) of a K^+^-functionalized Au electrode in response to varying concentration of K^+^. (**D**) Normalized values of measured *S*_11_ of a wireless K^+^ sensor. (**E** to **H**) Calibration plots for K^+^, Ca^2+^, Na^+^, and H^+^ wireless sensors in response to corresponding analytes.

Proper design of the sensing interface coupled to the LC circuit enables the capture, transduction, and readout of biochemical signals. The system successfully realizes the detection of multiple ions by using corresponding ISMs. The interface consists of a sensing electrode (SE) and a reference electrode (RE) (Fig. 3A). Both commercial bulk Ag/AgCl electrodes and self-made thin-film type ones (fig. S9) can serve as RE for stable signal readout. Figure 3B shows a photograph of a pair of thin-film electrodes (SE and RE). For the thin-film Ag/AgCl electrodes, it is crucial to encapsulate a layer of solid electrolytes consisting of KCl and polyvinyl butyral (PVB) for an improved stability against varying ion concentrations in the environment. As shown in fig. S9, varying the concentration of NaCl (5 to 80 mM) in the solution examines the stability of the two types of RE, suggesting the importance of having the encapsulation layer of solid electrolytes. The transport of ions from a high to a low concentration through a selective binding within the membrane creates a phase boundary potential according to the Nernst equation (Fig. 3A, right) (*42*). Biologically important ranges of ions in multiple biofluids [i.e., plasma, sweat, and cerebrospinal fluid (CSF)] reported in literatures (*43*–*49*) serve as reference data points when designing the experiments for this study. Detailed values/ranges appear in table S1. Figure 3C presents the measured open circuit potential (OCP) of a K^+^ ISM functionalized Au electrode (versus an Ag/AgCl RE). Ionophores are important for achieving a high specificity for sensing in complex environment with multiple ions. The structure of the K^+^ ionophore, valinomycin, appears in the inset. The alternating amino acids and esters in this cyclodepsipeptide form intramolecular hydrogen bonds, resulting in a cavity with the size matching the diameter of K^+^ (*50*). The system shows a near-Nernstian sensitivity (49.5 ± 1.5 mV/decade). When connected to the coupling unit, the surface potential difference serves as the reverse bias modulating the capacitance of the varactors, resulting in a shift in *f*_s_ (fig. S10). Figure 3D shows normalized data according to results in fig. S10. Extracted *f*_s_ as a function of K^+^ concentration appears in Fig. 3E, with a sensitivity of 2.7 MHz/decade. Following the same working principle, the wireless sensor system can selectively respond to other ions (Na^+^, Ca^2+^, and H^+^) in their biologically meaningful ranges via the integration of corresponding ISMs (Fig. 3, F to H, and fig. S11). The structures of the ionophores for Ca^2+^, Na^+^, and H^+^ are in the insets. The Ca^2+^ and Na^+^ ionophores interact with the corresponding ions in a way similar to valinomycin, while the basicity of amine in the H^+^ ionophore leads to its high affinity with H^+^ (*51*).

**Fig. 4.**
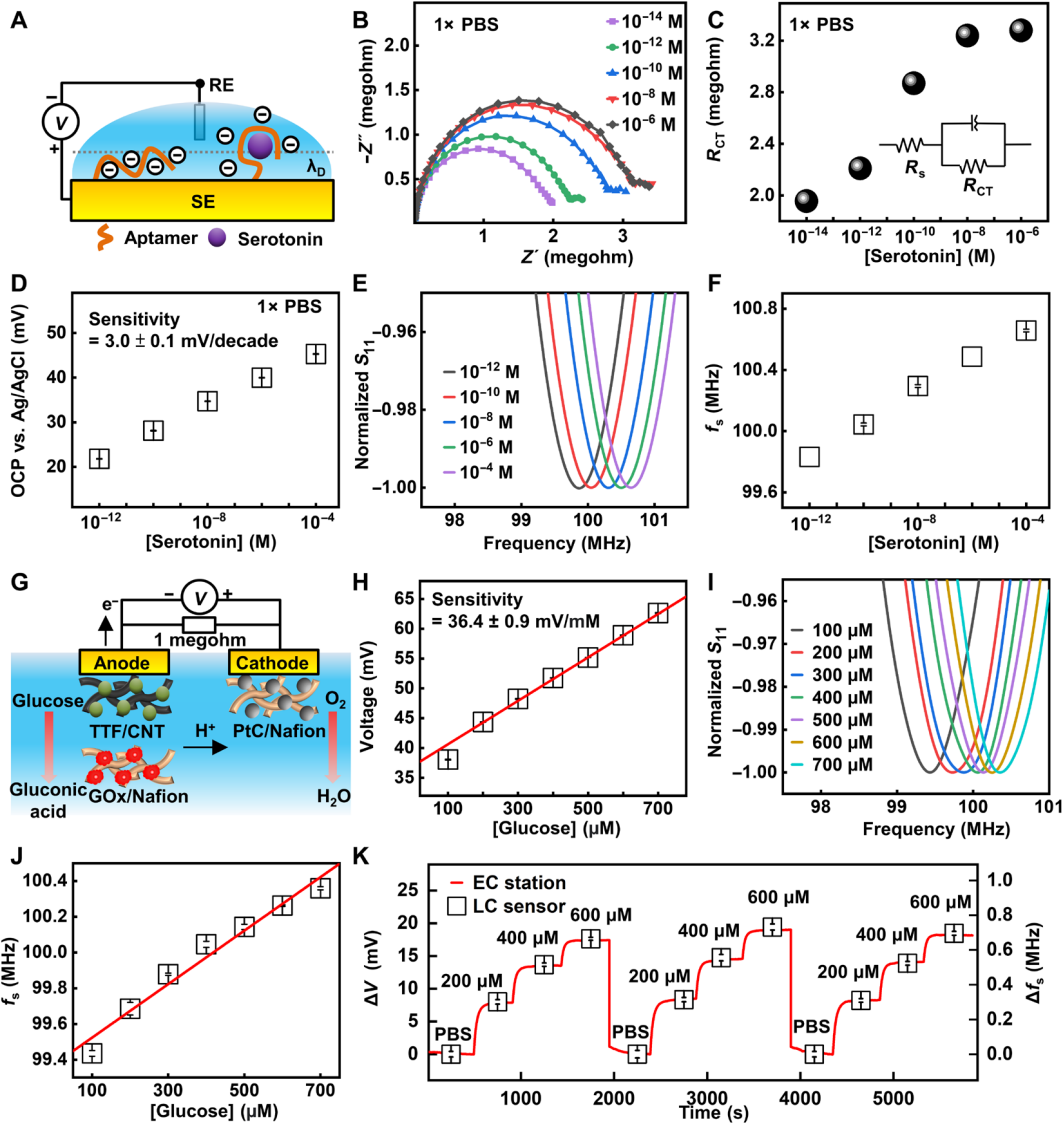
Design and performance characterization of aptamer- and enzyme-functionalized sensor systems. (**A**) Schematic illustration of the interaction between serotonin and an antiserotonin aptamer-functionalized Au surface. (**B**) EIS characterization of an aptamer-functionalized electrode in 1× PBS solutions with varying concentration of serotonin. (**C**) Charge transfer resistance extracted from EIS results in (B). (**D**) OCP (versus Ag/AgCl RE) of the sensing interface as a function of serotonin concentration in 1× PBS solution. (**E**) Resonance curves of a wireless sensor system integrated with antiserotonin sensing interface in response to serotonin in 1× PBS solutions. (**F**) *f*_s_ of the integrated sensor extracted from (E). (**G**) Schematic illustration of the biofuel cell–inspired biochemical interface for glucose sensing. (**H**) Measured voltage drop across the resistor connecting the cathode and anode of the sensing interface. (**I**) Resonance curves of a wireless sensor system integrated with biofuel cell–inspired sensing interface in response to glucose solutions. (**J**) *f*_s_ of the integrated sensor as a function of concentration of glucose. (**K**) Real-time data acquired with varying concentration of glucose measured simultaneously using an electrochemical station and a wireless sensor. The data during the three sensing cycles are collected separately with a pause between each cycle and plotted together for comparison.

Beyond ion sensing, the proper design of sensing interfaces with biorecognition elements enables the detection of various biomarkers. The device setup is the same when going from the ion sensors to the aptamer and glucose sensors reported in the following part, except that we changed the surface functionalization layer based on designs in the schematic illustrations. The use of aptamers prepared by the systematic evolution of ligands by exponential enrichment (SELEX) process can expand the applicability of the wireless sensors. Compared to ions with physiologically relevant concentrations at millimolar or micromolar level, the concentrations of some biomolecules (e.g., neurotransmitters and hormones) in bodily fluids are naturally much lower (typically at nanomolar or picomolar level) (*52*). As a result, minute concentration changes of these biomarkers sometimes fall below the limit of detection (LoD) and limit of quantification of conventional electrochemical sensors (*53*), when the Faradaic response is small compared to the background current. In this context, SELEX allows for the design of receptors to various analytes. The high affinity between the oligonucleotides and targets provides molecular recognition capability with high sensitivity and selectivity (*38*). As an example, Fig. 4A shows the schematic illustration of the interaction between serotonin, a key hormone for emotion regulation, and antiserotonin aptamers functionalized on an Au electrode surface. The binding event induces a conformational change of the DNA strand, resulting in a modification in surface potential within the Debye screening length (*38*), allowing for potentiometric sensing even in an environment with high ionic strength. Characterizations of the functionalized surface using electrochemical impedance spectroscopy (EIS) appear in Fig. 4 (B and C). The Nyquist plots exhibit an increased charge transfer resistance (*R*_CT_) between the redox probes ([Fe(CN)_6_]^3−^/^4−^) and the electrode surface upon the binding of more serotonin. The observation is likely due to the reorientation of negatively charged backbones of the aptamers away from the electrode surface that keeps redox probes even farther from the surface due to electrostatic repulsion. Figure 4D shows the OCP value of an aptamer-functionalized Au surface as a function of the concentration of serotonin in 1× phosphate-buffered saline (PBS) solution, with a sensitivity of 3.0 ± 0.1 mV/decade. For comparison, fig. S12 presents results obtained in 0.01× PBS, with a sensitivity of 3.6 ± 0.1 mV/decade. The LoD is ~10^−12^ M under both conditions. The increasing OCP values as serotonin concentration increases supports the reorientation of aptamer away from the surface, as the backbone of DNA carries negative charges, consistent with previous studies (*38*). The Debye length in 1× PBS and 0.01× PBS is ~0.75 and 7.53 nm, respectively, as calculated based on the definition (note S3) (*38*, *54*). For aptamers with 20 to 100 nucleotides, the length is around 2 nm (*55*). The antiserotonin aptamer used in this study has 57 nucleotides. Therefore, it is reasonable to estimate the length of the aptamer used here to be ~2 nm. The value is comparable to the Debye length in 1× PBS and 0.01× PBS. The binding event can induce a conformational change of the DNA strand, resulting in a modification in surface potential within the Debye screening length. On the basis of the same transduction principle, the surface potential can serve as the reverse bias driving the varactors for wireless, quantitative signal readout, with a sensitivity of 0.11 MHz/decade (Fig. 4, E and F). To further evaluate the feasibility of using the platform as biosensors, this study uses the data of the concentrations of the main ions present in sweat reported in literatures (*45*, *46*, *56*) to estimate the ionic strength of sweat (table S2). Results show that the value is in the range of 23.9 and 59.1 mM, which is lower than that of 1× PBS (162.7 mM) and higher than that of the 0.01× PBS (1.627 mM). Results obtained in solution with an ionic strength of 51.45 mM (~0.3× PBS) show a sensitivity of ~3.2 mV/decade and 0.096 MHz/decade (fig. S13), which is close to that obtained in 1× PBS. In addition to sensitivity, the study also evaluates the sensor performance in the presence of contaminants that could result in false positives or false negatives. Figure S14 presents the performance of antiserotonin aptamer–functionalized sensors in the presence of dopamine (1 ng/ml) and glutamate (1 μM). The sensor shows a monotonic increase in OCP in response to an increasing concentration of serotonin, and the extracted sensitivity is ~1.3 mV/decade. Comparison of the response of the antiserotonin sensor to serotonin and dopamine within the same concentration range in 1× PBS suggests the specificity (fig. S15). The sensor scheme in Fig. 4 (A to F) is the same as that in Fig. 3A except for the surface functionalization layer of SE. The reason for changing the functionalized interfaces is to incorporate the corresponding biorecognition elements for the selective detection of target analytes. The innovation is that the multiple designs described here allow for the detection of various biomarkers.

The wireless sensor is also compatible with detecting glucose when integrated with a biofuel cell–inspired sensing interface (*57*, *58*). Figure 4G illustrates the underlying sensing principle. The name means that the biofuel cell structure reported in the literature inspires the design of the sensor, although the purpose of using the biofuel cell in this work is for sensing instead of power supply: The anodic and cathodic reactions generate electrical currents proportional to the concentration of glucose. A load resistor (1 megohm) connecting the anode and cathode transforms the current into a voltage signal that the LC coupling unit can transmit. The relevance/importance of interface is that it allows for the detection of current generated by enzymatic catalysis by exploiting a biofuel cell structure and a load resistor. The working principle differs from that for the ISM- and aptamer-based sensors detecting changes in surface potential. Therefore, by using corresponding enzymes, the biofuel cell–inspired sensing interface further expands the categories of biomarkers that the sensor system can detect. Optimizing the structures of the cathode and anode yields sensors with a near-linear dynamic range encompassing the biologically meaningful concentration of glucose in sweat. Figure 4H presents the voltage across the resistor as a function of glucose concentration (sensitivity, 36.4 ± 0.9 mV/mM; LoD, 27 μM). The definition of LoD is 3.3 × *S*_r_/*S*, where *S*_r_ is the standard error of the calibration plot and *S* is the slope of the calibration plot. Figure 4 (I and J) and fig. S16 summarize the corresponding resonance curves and extracted *f*_s_ with a sensitivity of 1.5 MHz/mM. The real-time response to varying glucose concentration in a stepwise fashion (from 0 to 600 μM, reducing to 0 and repeating the process) appears in Fig. 4K. The results highlight the linear and reversible response with a minimal hysteresis to time-varying concentrations, which makes the system suitable for continuous monitoring of glucose in biofluids such as sweat and tears. The performance of the glucose sensor in commercial artificial sweat containing interferents such as amino acids, metabolites, and minerals appears in fig. S17, with an extracted sensitivity of 7.35 mV/mM and 0.44 MHz/mM. In addition, the sensor shows a negligible response to the addition of lactate (fig. S18). Those results demonstrate that the sensors have specificity to target biomolecules in mixed solutions. Customizing the system can enable the detection of different analytes using corresponding enzymes, such as lactate, ethanol, urea, and others. Together, the compatibility of this wireless sensor with various types of biochemical interfaces (ISM-, aptamer-, and biofuel cell–based) suggests the broad applicability of this device concept.

### Wireless sensor arrays for multiplexed chemical sensing

**Fig. 5.**
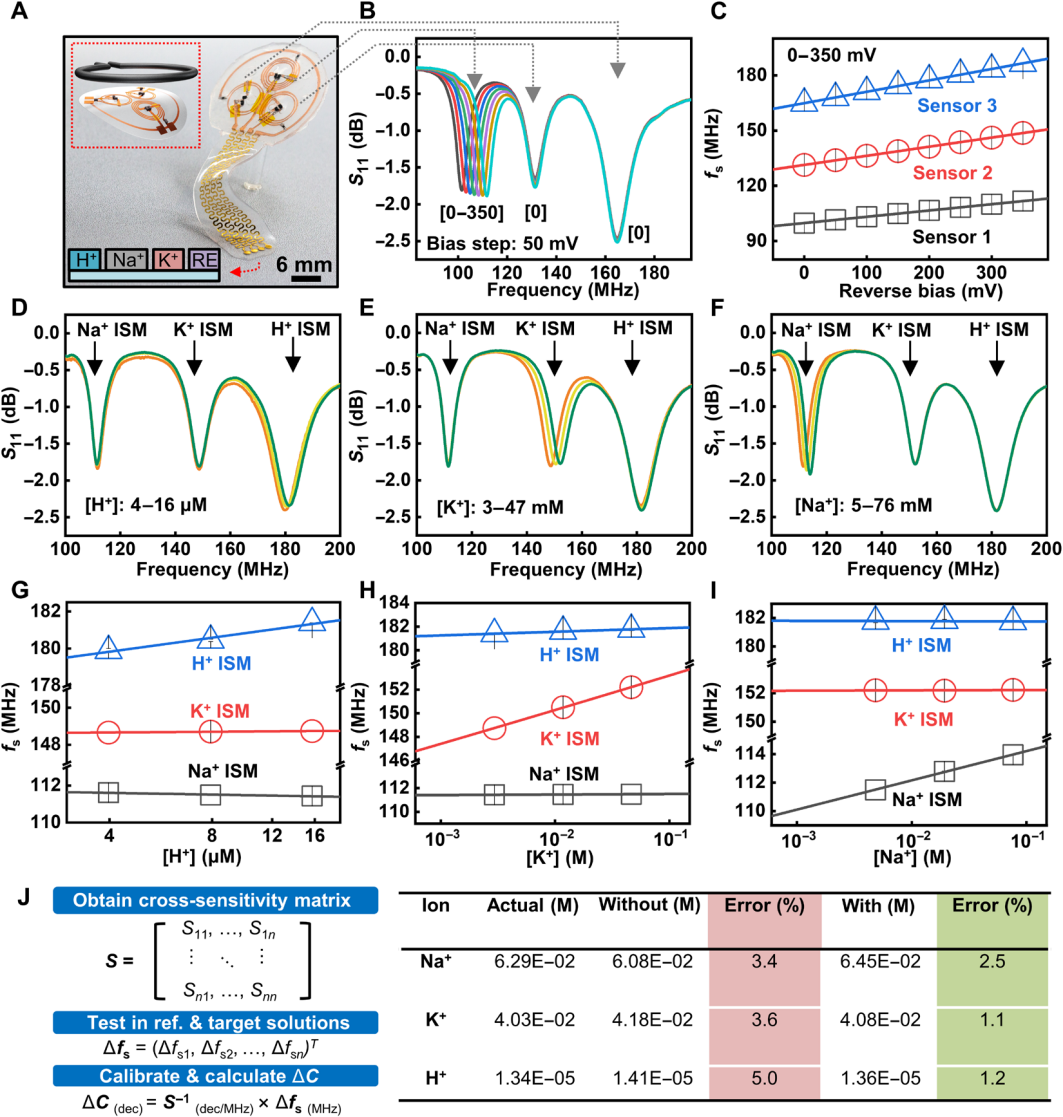
Multiplexed sensor system based on the LC circuit and sensing performance in mixed ion solutions. (**A**) Photograph of a multiplexed ion sensor system consisting of three LC circuits with varied resonance frequency. The four electrodes (from left to right) correspond to H^+^, Na^+^, and K^+^ sensors and the RE. (**B**) Resonance curves of the multiplexed sensor array with a reverse DC bias applied to one LC circuit. (**C**) Response to input DC voltage of the three sensors in the multiplexed sensing platform. (**D** to **F**) Resonance curves of the multiplexed sensing system in response to concentration change in H^+^, K^+^, and Na^+^ in the mixed ion solution. (**G** to **I**) Extracted cross-sensitivity of the multiplexed sensing system based on results in (D) to (F). (**J**) Calibration methodology used for the multiplexed sensor arrays and calculated ion concentrations in a mixed ion test solution obtained without and with calibration.

Another important performance metric of biochemical sensors is multifunctionality, which allows for the simultaneous detection of multiple biomarkers with a minimum level of cross-talk. These multiplexed sensing platforms can support the capture of biometric signature profiles for dynamic, personalized, and adaptive treatments. Designing coils with varied inductance yields well-separated *f*_s_. As shown in Fig. 5 (A and B), a two-, three-, and four-turn coil (diameter, 10 mm) has a *f*_s_ of 160, 130, and 100 MHz, respectively. The four electrodes in the DC part (from left to right) correspond to H^+^, Na^+^, and K^+^ sensors and a shared Ag/AgCl RE. Varying the input voltage to one coupling unit induces shift in *f*_s_ accordingly, while the values of *f*_s_ of the other two stay almost constant (Fig. 5, B and C). Similarly, applying a reverse bias voltage to the other two sensors in the system induces shifts in *f*_s_ of the corresponding devices with a near-linear voltage response, as shown in fig. S19. On the basis of the frequency modulation sensing mechanism, the multiplexed sensors allow for simultaneous readout of multiple biochemical signals within a single scan.

In a mixed solution, the concentration change in each ion can induce a slight change in the resonance frequency of other sensors because of the nonspecific interactions between ISMs and nontarget ions. Therefore, one ion concentration change will affect another ion concentration sensing. When two (or more) ion concentrations vary at the same time, the signal change in each sensor is a result of the combined response to all ions within the solution (fig. S20). This study reports a calibration method to address the issue: Systematically varying the composition of a mixed solution ([H^+^] = 4 μM, [Na^+^] = 5 mM, and [K^+^] = 3 mM) and characterizing the response allow for characterization of the specific and nonspecific interactions within the system (Fig. 5, D to F). The concentration variation of the base solution takes place according to the following order: [H^+^] from 4 to 16 μM, [Na^+^] from 5 to 76 mM, and [K^+^] from 3 to 47 mM. Summary of the cross-sensitivity of each sensor to different ions appear in Fig. 5 (G to I). The results suggest that each sensor shows a response with the variation in concentration of both the target and nontarget ions. Extracting the values of cross-sensitivity establishes the sensitivity matrix for signal calibration. For example, when the concentration of H^+^ changes (Fig. 5D), the shift in the curves for Na^+^ and K^+^ sensors are due to nonspecific interactions between Na^+^ and K^+^ ISMs and H^+^. Extracted *f*_s_ of the three sensors during the cross-sensitivity test in mixed ion solutions are in table S3. As shown in this table, the shifts due to nonspecific interactions in Fig. 5 (D to F) are at a similar order of magnitude in *f*_s_ (all less than 0.5 MHz). The sensitivity matrix allows for the calibration of the sensing results with improved detection accuracy according to the Nikolskii-Eisenman equation (*59*). Briefly, the model assumes that the response of a sensor in a complex environment is the sum of responses to specific (*S_ii_*) and nonspecific (*S_ij_*, *i* ≠ *j*) interactions, and calibration using the sensitivity matrix can separate the two types of signals (Fig. 5J, note S4, and fig. S21). Figure 5J (right) shows the actual concentrations of three ions in a mixed solution and the calculated results before and after calibration. In all cases, the use of the sensitivity matrix for calibration reduces the value of error (H^+^, from 5.0 to 1.2%; Na^+^, from 3.4 to 2.5%; and K^+^, from 3.6 to 1.1%). The results presented here suggest that using the multiplexed sensing platform with the calibration standards can provide an improved accuracy for chemical sensing in complex environment.

### Demonstrations of the wireless sensor prototype as biointegrated electronics

**Fig. 6.**
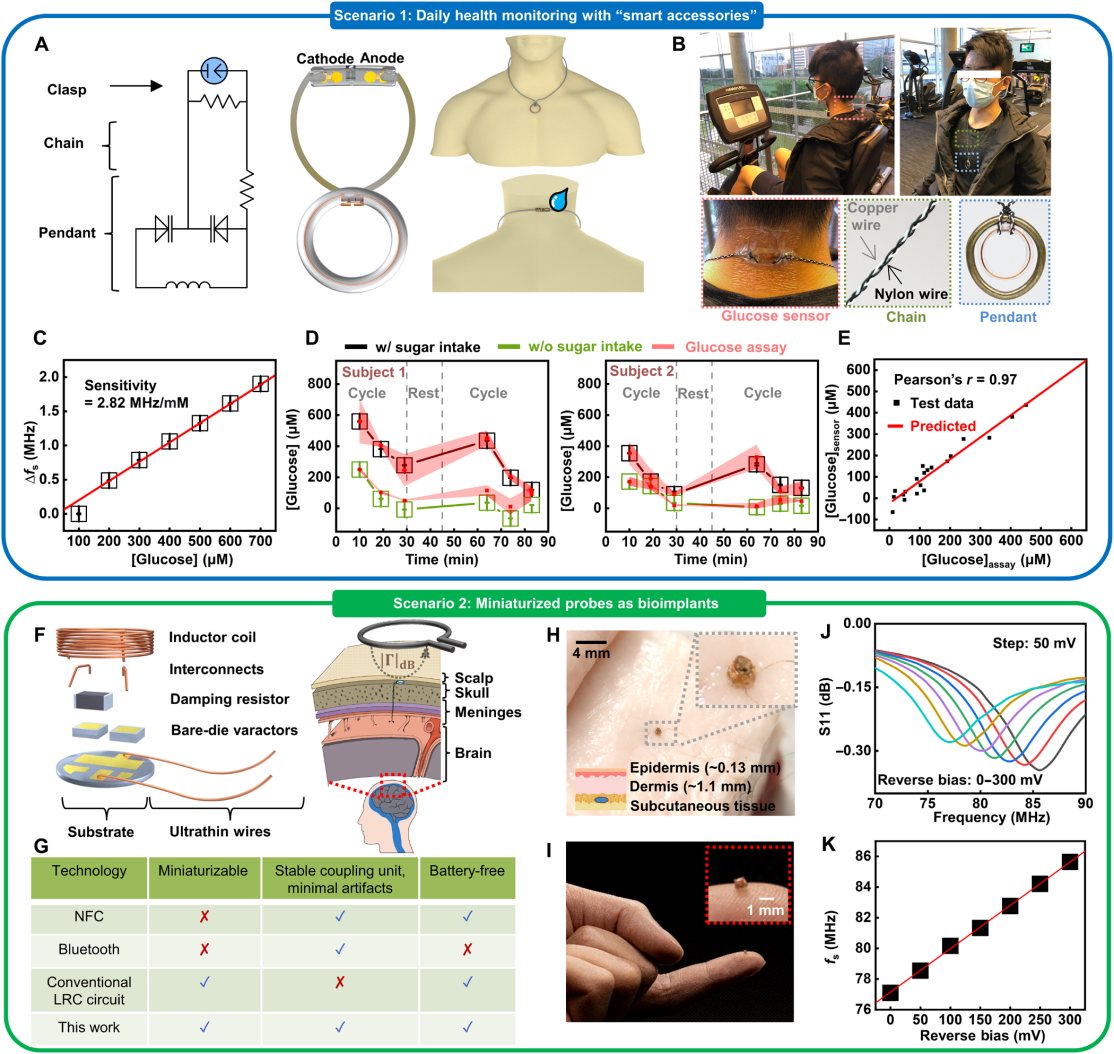
Demonstrations of biointegrated chemical sensors customized for different application scenarios. (**A**) Design and schematic illustration of a smart necklace based on the tuning circuit–inspired sensor prototype, including a pendant, a clasp, and a chain. (**B**) Photographs of a participant wearing the smart necklace for sweat analysis during cycling. (**C**) Calibration plot of the smart necklace used for field testing. (**D**) Real-time wirelessly acquired signals and the corresponding data obtained using a commercial glucose assay kit in two participants during cycling, showing changes in glucose concentration in sweat. (**E**) Comparison of glucose concentrations measured by the smart necklace and the commercial assay kit. The data are from 20 sweat samples shown in (D), excluding the first point of each study section used for baseline correction. (**F**) Schematic illustration of a miniaturized sensor probe based on the resonance circuit for potential applications as bioimplants. (**G**) Comparison between the miniaturized sensor probe and competing state-of-the-art wireless sensing technologies. (**H** and **I**) Photographs of a miniaturized coupling unit mounted on a fingertip and integrated with a piece of meat, respectively. (**J** and **K**) Electrical performance of a miniaturized probe with an input reverse bias simulating biochemical signals.

As mentioned in the preceding section, a key and unique feature of this tuning circuit–inspired sensor system is that it consists of an AC part for wireless signal transmission and DC part for interfacing target biofluids. Customizing this modularized circuit model can form various biointegrated sensor systems suitable for different application scenarios (Fig. 6). In the first example, the battery-free sensor prototype allows for easy integration with personal accessories due to the light weight and the simple circuit layout, providing strategic advantages in health monitoring during daily activities, as it does not require the use of on-chip integrated circuits. As a proof of concept, the design and schematic illustration of a smart necklace appear in Fig. 6A and fig. S13. Figure S22 presents the optical image of the structure of the sensing interface. Encapsulating the sensing interface and the recyclable coupling unit into epoxy scaffolds forms the clasp (placed on the back of the neck) and pendant parts, respectively. Here, the clasp includes a pair of functionalized cathode and anode for glucose sensing. Conductive, stretchable wires serve as the chain connecting the clasp and pendant. The copper wires have an encapsulation layer to prevent electric shock. Following the same circuit model, a resistor (incorporated in the pendant) in series with the sensing interface can isolate the DC part and minimize motion and/or environment-induced changes in *f*_s_ (e.g., deformation/stretching of the chain). Field testing involving healthy, consenting human volunteers demonstrates the practical application of the smart necklace. Figure 6B shows a human participant wearing the sensor and the key functional parts of the necklace. Aligning a single turn readout coil connected with a portable VNA to the pendant records the stimulus spectrum within ~10 s (fig. S13). The sensor reported here measures the change in surface potential, which scales with the change in chemical concentration of analytes. Therefore, the calibration of the baseline at the beginning of each study is important for obtaining the actual values of glucose in target biofluids, because factors such as the health/skin conditions of the participant and the quality of the enzyme-functionalized sensing interface can affect the absolute values of *f*_s_ during different study sections. To address this issue, the study calibrates the sensor and confirms that it has a linear sensitivity throughout a broad concentration range (2.82 MHz/mM; Fig. 6C). During the field testing, collecting sweat samples simultaneously and using the first data point of glucose concentration determined by the commercial assay kit (collected after 10 min) establish the baseline for calculating the sensing results for the rest part of the study. Figure 6D shows a summary of data acquired from two participants. Here, tests start with the participant wearing the “necklace” and cycling ~30 min to generate sweat. Then, the participants take a 15-min break, drink sugar-sweetened beverages (150 ml; ~17 g of sugar), and resume cycling. The results show that, in all cases, the glucose concentration in sweat reaches a peak within ~30 to 40 min after the sugar intake. The concentration then drops because of a combined effect of glucose-induced insulin release and exercise. The effect of exercise may also explain the variation during the predrinking cycle. Experiments following the same protocols but without drinking sweetened beverages examine the effect of sugar intake. The results suggest a less-obvious spike in glucose concentration afterward, which indicates that drinking sugar can induce an increase in the amount of glucose in sweat. Comparison of the sensing results with those determined by commercial assay kits validates the accuracy of this sensor for analyzing sweat samples [Pearson correlation coefficient (*r*) = 0.97] (Fig. 6E). Please note that, when the difference between the baseline sample and the test sample is very close to the absolute value of the former, calculations may yield slightly negative results. Further enhancing the precision in characterizing the sensitivity of sensors may address this issue for future applications. Overall, the results suggest the potential of using the “smart accessories” as a noninvasive means for tracking biomarkers in sweat. In addition, designing the sensor system in a detachable layout with a recyclable coupling unit and a replaceable sensing interface could provide a viable solution for future remote health care due to the reusability and low-cost nature.

The study also demonstrates the feasibility of developing miniaturized sensing probes for potential applications as bioimplants in the future. Decapping commercial varactor diodes minimizes the size of the system. Wrapping a pair of diodes with ultrathin conductive wires forms the inductor coil. Figure 6F presents the exploded view of an as-prepared probe and a conceptual illustration of a potential application where the coupling unit is embedded in the skull to provide stable signal transmission, while the sensing interface extends to the area with CSF or brain tissues of interest. Comparison of this platform with other competing technologies appears in Fig. 6G: Conventional resonance circuit–based biochemical sensors typically have a functionalized electronic component integrated within the LC tank, the resistance/capacitance of which can be modulated upon chemical adsorption. However, the part interfacing with biotissues may affect the performance of the electromagnetic coupling unit and, thus, can frustrate the quantitative interpretation of biochemical signals. In our system, the separation of the sensing interface from the electromagnetic coupling unit can enable the placement of the AC and DC parts into different locations with ultrathin wires as interconnects and the resistor minimizing motion/environment-induced artifacts in the DC part. Moreover, compared with the state-of-the-art Bluetooth and Near-field communication (NFC) wireless schemes, the system minimizes the use of rigid components, which substantially improves the conformability for biointegration. Figure 6 (H and I) shows an as-prepared coupling unit with a diameter of ~1 mm mounted on a fingertip and integrated with a piece of meat, respectively. The coupling unit shows that the resonance frequency shifts as a function of a DC signal input bias voltage simulating biochemical signals with a similar level of amplitude (~50 to 300 mV) (Fig. 6, J and K). The miniaturized probe, when combined with the versatile sensing interface described in Figs. 3 and 4, provides a promising strategy for building implantable biochemical sensors.

## DISCUSSION

In summary, the materials, electronic designs, and integration schemes presented in this work provide a promising route to developing miniaturized, lightweight, wireless, and biointegrated technologies. The resulting system combines LC resonance circuit, varactor diode, stretchable design, and biorecognition elements for detection of biological markers in bodily fluids. Inspired by the working principle of tuning circuits in RF electronics, the coupling between LC circuits and sensing interfaces through a pair of varactor diodes enables battery-free operation and accurate monitoring of multiple biomarkers with a simple circuit layout. Specifically, the use of varactor diodes modularizes the system into an AC part (coupling unit) that can be encapsulated separately and a DC part (sensing interface) in contact with target biofluids. The integration of a series resistor with the DC part can minimize strain-induced changes in electrical properties of the circuit, enabling the high-fidelity recording in the presence of external strains. Demonstrations of sensors with ISM-, aptamer-, and enzyme-functionalized interfaces suggest that this sensing platform can support the detection of ions, neurotransmitters, and metabolites via proper design of the surface structure and coupling strategy. Human participant studies with a smart necklace built on the circuit model demonstrate that these devices can implement robust signal recording and data transmission without failure during physical exercise. Overall, the sensing platform provides strategic advantages for recording, tracking, and understanding the body’s dynamic chemistry. Devices of this type can serve as a complementary tool for health status tracking and closed-loop health management to commercial wearable sensors that record biophysical signals. By wirelessly tracking biomarker concentrations associated with the response of the body to environment, stress, and disease, this innovative, versatile device concept can find broad applications in biomedical research and clinical practices. Immediate opportunities are with developing lightweight, miniaturized, and portable readout circuits to replace the commercial bulky VNA for data transmission to user interfaces, which can be easily integrated with the user’s personal belongings, such as clothes and cell phones for remote health monitoring (*32*).

## MATERIALS AND METHODS

### Fabrication of LC circuit–based sensing platform

The fabrication of the electromagnetic coupling unit started with laminating a conductive copper tape (adhesive side facing up) on an ultraviolet (UV) light dicing tape. Cutting the copper tape using a vinyl cutter (Silhouette Cameo 4) followed by exposing the system to UV light and peeling off unneeded parts of the tape yielded patterned conductive traces. Pasting the adhesive side of the copper tape on a polyester (PET) film and removing the UV dicing tape finished the transfer process. Soldering a pair of varactor diodes (SMV1249-040LF, Skyworks Solutions, Inc., CA, USA) and a 10-kilohm resistor to the metal traces formed the LC circuit. The preparation of the electrodes as the sensing interface followed the same procedures, except that Cr (10 nm)/Au (300 nm) deposited on a polyimide (PI) film (~13 μm) using electron beam evaporation served as the conductive metal traces that provided stable signal readout because of the chemical inertness of Au. Connecting the DC and AC components using silver epoxy completed the fabrication of the stretchable electronic device.

### SPICE and near-field simulation

The SPICE simulation of the reflection response of the equivalent circuit at different frequencies was performed using the analog electronic circuit simulator (LTspice, Analog Devices). Small signal (linear AC) analysis determined *S*_11_ and resonance frequency.

A commercial electromagnetic simulator (Feko, Altair) performed the near-field simulation. The simulation captured the inductive coupling behavior between the LC tank (with a three-turn coil; diameter, 10 mm) and the readout coil. Key parameters used were as follows: A 20-pF capacitor was connected to the three-turn coil to form the LC circuit. The single-turn readout coil (diameter, 10 mm) was stimulated with an incident AC voltage source (1 V; 50 to 200 MHz). The distance of the gap between the LC tank and readout coil was set to fixed values (3 to 13 mm).

A commercial finite element analysis simulator (COMSOL Multiphysics) performed the simulation to estimate the SAR and heat transfer of the system with a tissue model. The simulation studied how a tissue absorbs RF energy radiated from a single-turn coil antenna (diameter, 10 mm) with an incident AC power source (−9 dBm, 100 MHz). The simulation defined the antenna as a perfect electrical conductor. The distance between the antenna and tissue surface was 0.5 mm.

### Preparation of ISM-based sensing interfaces for Na^+^, K^+^, Ca^2+^, and H^+^

The preparation of polymeric ISMs followed steps reported in previous publications (*60*). The recipe for each membrane is as follows: (i) Na^+^-ISM: sodium ionophore X (1 wt %), sodium tetrakis[3,5-bis(trifluoromethyl)-phenyl] borate (Na-TFPB; 0.55 wt %), polyvinyl chloride (PVC; 33 wt %), and bis(2-ethylhexyl) sebacate (DOS; 65.45 wt %). Dissolving 100 mg of the mixture in 660 μl of tetrahydrofuran formed the Na^+^ ISM cocktail. (ii) K^+^-ISM: valinomycin (2 wt %), sodium tetraphenylborate (0.5 wt %), PVC (32.7 wt %), and DOS (64.7 wt %). Dissolving 100 mg of the mixture in 350 μl of cyclohexanone formed the K^+^ ISM cocktail. (iii) Ca^2+^-ISM (directly purchased from Sigma-Aldrich): calcium ionophore IV (0.072 wt %), Na-TFPB (0.022 wt %), 2-nitrophenyl octyl ether (4.748 wt %), PVC (2.379 wt %), and tetrahydrofuran (92.78 wt %). (iv) H^+^-ISM: hydrogen ionophore I (1 wt %), sodium tetrakis(4-chlorophenyl)borate (Na-TC|PB; 0.65 wt %), PVC (33 wt %), and DOS (65.35 wt %). Dissolving 100 mg of the mixture in 660 μl of tetrahydrofuran formed the H^+^ ISM cocktail. Casting the mixtures on an Au surface and drying the system overnight completed the preparation of ISM-functionalized sensing interface.

### Preparation of thin-film Ag/AgCl RE

The preparation of the RE started with drop-casting a mixture of silver epoxy and hardener (Chemtronics CW2400) on an Au electrode surface and curing it at room temperature for 12 hours. Treating the electrode with sodium hypochlorite solution (5 wt %) for 30 min converted the surface to AgCl. In a separate process, recrystallizing KCl_(aq)_ in cold isopropyl alcohol formed ultrafine microsized powders. Dissolving 438 mg of PVB (10 wt %) in 5 ml of anhydrous ethanol, mixing the solution with 250 mg of KCl powder, and homogenizing the system in an ultrasonic bath for 10 min yielded an electrolyte cocktail (stored at 7°C). Casting the electrolyte cocktail on the Ag/AgCl electrode and drying the whole system overnight completed the fabrication of the RE.

### Preparation of aptamer-based sensing interface for serotonin

Thiolated, single-stranded antiserotonin DNA aptamer [5′ to 3′: HO(CH_2_)_6_-S-S-(CH_2_)_6_-CTC TCG GGA CGA CTG GTA GGC AGA TAG GGG AAG CTG ATT CGA TGC GTG GGT CGT CCC] was purchased from Integrated DNA Technologies (Coralville, IA). The DNA aptamer received from the manufacturer was in an oxidized state with S atoms protected in the form of disulfide bonds. Reducing the oxidized DNA aptamer with dithiothreitol (DTT) [100 μM DNA aptamer and 10 mM DTT in 1× tris-ethylenediaminetetraacetic acid (TE) buffer] followed by removing DTT through centrifugation in Macro SpinColumns (Harvard Apparatus) yielded DNA aptamer with thiol groups on the 5′ ends for binding to Au surfaces. Mixing the aptamer (50 μM) and 6-mercapto-1-hexanol (MCH) (1:100) in 1× TE buffer formed the coating solution for the Au surface. Before functionalization, treating the Au electrode by cyclic voltammetry in 0.5 M H_2_SO_4_ (−0.4 to 1.5 V versus Ag/AgCl) for three cycles cleaned the surface. Drop-casting the mixture onto the cleaned Au electrode and drying the system at room temperature overnight formed Au─S bonds and, thereby, completed the functionalization. Before testing, immersing the SE in 1 mM MCH solution for 30 min further passivated any remaining exposed Au area. After an incubation period of 30 min in PBS solutions with different concentrations of serotonin, an electrochemical workstation (PalmSens4, CA, USA) recorded the OCP (versus Ag/AgCl RE) with a sampling rate of 1 Hz. The characterization of EIS used a three-electrode setup with a Ag/AgCl RE and a platinum wire counter electrode. K_4_Fe(CN)_6_/K_3_Fe(CN)_6_ (1,1) (2 mM) served as the redox couple. Simulation of the measured Nyquist plots using the Z-view software based on a Randles circuit determined the charge transfer resistance (*R*_CT_).

### Preparation of the biofuel-based sensing interface for glucose

The preparation of biofuel cell–based sensors followed previous studies (*57*, *58*). The enzyme-modified anode consisted of tetrathiafulvalene (TTF), GOx, bovine serum albumin (BSA), and Nafion 117. Dissolving TTF (Sigma-Aldrich, MO, USA) in a mixture of ethanol/acetone (9:1, v/v) and homogenizing it in an ultrasonic bath for 10 min formed uniform solution (0.1 M). Drop-casting 32 μl of TTF solution onto a multiwalled carbon nanotube (MWNT) paper (100 mm^2^; Buckypaper, GSM 20, NanoTechLabs, NC, USA) and letting dry for 1 hour under ambient condition formed TTF-modified carbon nanotube pads. Dispersing GOx (40 mg/ml; Sigma-Aldrich, MO, USA) and BSA (10 mg/ml; Sigma-Aldrich, MO, USA) in 1× PBS and 1× PBS with Nafion 117 (Sigma-Aldrich, MO, USA) (1 wt %), respectively, followed by mixing the two suspensions in equal volumes, yielded the enzyme-coating suspension. Applying 64 μl of the enzyme-coating suspension onto the TTF-treated MWNT paper, letting dry for 1 hour under ambient condition, and storing it at 7°C for a week allow the system to equilibrate. Last, using a mechanical puncher to cut an enzyme-functionalized pad (diameter, 1 mm) and bonding it to a gold current collector with silver epoxy completed the fabrication of the anode.

The preparation of the cathode started with dispersing platinum on carbon (Pt/C, 10%; Sigma-Aldrich, MO, USA) in a Nafion solution (2 wt % in ethanol). Applying 5 μl of the resulting suspension (10 mg/ml in Nafion solution) on a MWNT paper (1 mm^2^), drying the solution under ambient condition for 1 hour, and storing it at 7°C for a week formed the functionalized cathode. Bonding the Pt/C-modified pad to a gold current collector with silver epoxy completed the fabrication. Exposing both the cathode and anode to 1× PBS for 30 min before testing allowed the system to reach equilibrium, providing stabilized signals.

The selectivity study used commercial artificial sweat (Pickering Laboratories Inc., Artificial Eccrine Perspiration, stabilized, NC9953115) as the base. Adding different amount of glucose into the system formed a set of test solutions.

### Fabrication of smart necklace and miniaturized probe

The fabrication of the pendant in smart necklace followed the same steps for preparing the LC circuit, as detailed in the preceding section. Encapsulating the LC circuit with waterproof clear epoxy formed the pendant with two exposed contact pads. Weaving a flexible conductive copper wire with two strings of nylon wire yielded the chain. Depositing Cr (10 nm)/Au (300 nm) on a PI film (~13 μm) and transferring it onto a PET film formed the current collectors for the clasp. Subsequently, bonding an enzyme-modified MWNT pad and a Pt/C-modified pad to the corresponding current collectors formed the anode and cathode, respectively. A 1-megohm load resistor was bonded to the Au traces by silver epoxy. Last, electrically connecting the pendant, chain, and clasp via soldering followed by encapsulating the junctions with waterproof epoxy completed the fabrication of the smart necklace.

The fabrication of the miniaturized probe started with depositing metal traces [Cr (10 nm)/Au (300 nm)] onto a PET film as electrical interconnects using electron beam evaporation and a shadow mask. A mechanical puncher defined the 1-mm PET pad as the substrate for the coupling unit. Bonding a pair of decapped bare-die varactor diodes (SMV1249, Skyworks Solutions, Inc.), a thick-film resistor (10 kilohms, imperial code = 01005), jumping wires (Cu, wire diameter = 50 μm), and a manually wrapped inductor coil (Cu; coil diameter = 1 mm, 11 turns, wire diameter = 50 μm) to the deposited metal traces formed the miniaturized LC circuit. Encapsulating the whole device with waterproof UV-curing resin (NOA 61, Norland Products, USA) completed the fabrication.

### Establishing the calibration plot with a commercial glucose assay kit

Mixing the test solutions (sweat samples) (20 μl) and colorimetric assay reagent [100 μl; Glucose (HK) Assay Kit, Sigma-Aldrich] followed by incubation at room temperature for 15 min allowed reactions to complete within a 96-well plate. A microplate reader (SpectraMax M3, Molecular Devices) measured the absorbance value at 340 nm. Calibrating the results with standard references determined the concentration of test solutions.

### Data collection and analyses

The readout circuit consisted of a single turn coil connected to a portable VNA (NanoVNA) through SubMiniature version A connectors. Setting the VNA to reflective mode enabled the measurement of the real and imaginary part of the reflection coefficient. Expressing the raw data in decibels yielded *S*_11_. Sweeping the frequency range obtained the attenuation of the coupling unit around *f*_s_. For the characterization of a single sensor system, sweeping a frequency range of 20 MHz at a step of 19.82 kHz per step obtained 1010 data points for the resonance curve, with a measurement time of ~8 s and a sampling rate (for obtaining one resonance curve) of 0.125 Hz. The study exploited OriginPro for the postprocessing of the raw data obtained by the VNA: A low-pass fast Fourier transform algorithm removed the background thermal noise and smoothed the resonance curve. The minimum peak finding function of the software identified *f*_s_. To allow for comparison of *f*_s_ among different resonance curves, dividing each data point in the curve by the absolute value of the peak normalized the raw data and set the peak value of each curve to −1.

### Human participant studies

Informed written consent was obtained before the field testing with human participants recruited from laboratory staff. The study was performed in compliance with all ethical regulations under a protocol approved by the Institutional Review Board at the Ohio State University (study number: 2021H0205).

## Supplementary Materials

### This PDF file includes:

Tables S1 to S3 Notes S1 to S4 Figs. S1 to S22
