# Supplementary material for: Battery-free, tuning circuit–inspired wireless sensor systems for detection of multiple biomarkers in bodily fluids
Source: Sci Adv. 2022 Jul 6;8(27):eabo7049. doi: 10.1126/sciadv.abo7049 (PMC9258955; doi:10.1126/sciadv.abo7049)
Supplement: Supplementary file 1 — Tables S1 to S3 Notes S1 to S4 Figs. S1 to S22 [file sciadv.abo7049_sm.pdf]

Science Advances
AAAS

Supplementary Materials for

Battery-free, tuning circuit–inspired wireless sensor systems for detection of multiple biomarkers in bodily fluids

Tzu-Li Liu *et al.*

Corresponding author: Jinghua Li, li.11017@osu.edu

Sci. Adv. **8**, eabo7049 (2022)
DOI: 10.1126/sciadv.abo7049

This PDF file includes:

Tables S1 to S3
Notes S1 to S4
Figs. S1 to S22

Table S1. Biologically significant ranges of ions investigated in this study based on reports in literature.

Biofluids	Na^+ (mM)	K^+ (mM)	Ca^{2+} (mM)	pH	H^+ (mM)	References
Plasma	142	5	2.5	7.38-7.42	~4.2×10^{-5}	(*43*), (*44*)
Sweat	42.9 ± 18.7	6.0 ± 0.9	0.375	~ 6.3 (mean value)	5×10^{-4}	(*45*), (*46*), (*47*)
Cerebrospinal fluid	147-150.1	2.8-3	1.15-1.3	~ 6.3	5×10^{-4}	(*48*), (*49*)

Supplementary Note S1
Plotting of the calibration curve

The coupling between the sensing interface and the varactor diodes converts the change in surface potential into a modification in capacitance and then into a shift in f_s according to the following equation:

$$f_s = \frac{1}{2\pi\sqrt{LC}} \qquad \text{(S1)}$$

where f_s is the resonance frequency, and L and C are inductance and capacitance of the resonance circuit, respectively. According to the datasheet of the varactor diode (SMV1249, Skyworks), the capacitance C_T as a function of the reverse bias (V_R) can be calculated using the following equation (Fig. S2B):

$$C_T\,(V_R) = \frac{C_{j0}}{\left(1+\frac{V_R}{V_J}\right)^M} + C_P \qquad \text{(S2)}$$

where V_J is the junction potential, M is the grading coefficient, C_{J0} is the zero-bias junction capacitance, C_P is the package capacitance, and V_R is the reverse bias applied to the varactor. The values used for the calculation are as follows: $V_J = 80$ V, $M = 70$, $C_{J0} = 36.4$ pF, $C_P = 1.68$ pF. Combining Equation S1 and S2 and taking the first derivative yield the slope of $f_s\,(V_R)$:

$$\frac{df_s(V_R)}{dV_R} = \frac{1}{2\pi\sqrt{L_T}} \times -0.5 \times C_T(V_R)^{-1.5} \times \frac{-M \times C_{j0}/V_J}{\left(1+\frac{V_R}{V_J}\right)^{(M+1)}} \qquad \text{(S3)}$$

when V_R is very small (e.g., < 1000 mV) compared to V_J (80 V), the slope can be viewed as a constant (Fig. 1F). The experimental results agree well with the plot derived by combining equation S1 and S2 (obtained using Matlab®) (Fig. 1F and S2A), showing the near-linear relationship between V_R and f_s in the range of low bias voltage relevant to biochemical sensing described in this work.

Supplementary Note S2
Fabrication of SiGe/Si pn diodes

The SiGe/Si pn diode fabrication began with the commercial epitaxial growth of a ~1 mm-thick n+ layer (phosphorous doped, 2-3 $\times 10^{19}$ cm^{-3}) of Si, a ~0.5 mm-thick n- layer (phosphorous doped, 4-5 $\times 10^{16}$ cm^{-3}) of Si, and a 30 nm-thick p+ layer (boron doped, 1 $\times 10^{20}$ cm^{-3}) of $Si_{0.74}Ge_{0.26}$ on a high-resistivity p- type Si substrate using an ultra-high vacuum chemical vapor deposition (UHVCVD) system. The Ge profile in the $Si_{0.74}Ge_{0.26}$ (SiGe, ibid.) layer is box type. The use of SiGe instead of Si as the p layer is to reduce the pn junction built-in potential (diode turn-on voltage). Using photoresist as mask to protect the SiGe/n-Si layer, circular mesa patterns with a diameter of 500 mm were formed by dry etching using a Reactive Ion Etcher (Plasma-Therm 790 Series). After the n+ Si layer was exposed, the photoresist was removed. Circular anode metal patterns with a diameter of 420 mm and ring-shaped cathode metal patterns with inner/outer diameters (580/620 mm) were formed on the anode SiGe mesa and the cathode n+ layer, respectively, using optical photolithography and liftoff techniques. Both the anode and the cathode metal stacks consist of Ti/Au: 50/100 nm. The anode can be probed directly and the cathode can be probed on the metal pads that connect the ring-shaped cathode.

Supplementary Note S3
Calculation of the Debye length and ionic strength of a solution

The following equation defines the Debye length λ_D of a solution:

$$\lambda_D = \sqrt{\frac{\varepsilon_0 \varepsilon_r T K_B}{2Ie^2 N_a}} \quad \text{(S4)}$$

where ε_0 is the permittivity of free space, ε_r is the dielectric constant of the solution, T is the temperature (Kelvin), K_B is the Boltzmann's constant, I is the ionic strength of the solution, e is the elementary charge, N_a is Avogadro's number. Based on the equation, the Debye length in 1X PBS and 0.01X PBS is ~0.75 and 7.53 nm, respectively (*38*) (*54*).

The following equation defines the ionic strength I of a solution based on the ion concentration(s):

$$I = \sum_{i=1}^{n} c_i z_i \quad \text{(S5)}$$

where c_i is the molar concentration of the ion i, and z_i is the charges carried by the ion i. Therefore, the ionic strength of the 1X PBS and 0.01X PBS solution is 162.7 and 1.627 mM, respectively.

We use the data of the concentration of the major ions in sweat from literature to estimate the ionic strength of sweat.

Table S2. Concentrations of major ions presenting in sweat reported in literature.

**Ion**	**Concentration mmol/L**	**References**
Na^+	42.9 ± 18.7	(*45*)
K^+	6.0 ± 0.9	(*45*)
Cl^-	32.2 ± 15.6	(*45*)
Ca^{2+}	0.375	(*46*)
Mg^{2+}	0.054	(*46*)
Zn^{2+}	0.006	(*56*)
Cu^{2+}	0.013	(*56*)
Fe^{3+}	0.018	(*56*)

Results show that the value is in the range of 23.9 and 59.1 mM, which is lower than that of 1X PBS (162.7 mM) and higher than that of the 0.01X PBS (1.627 mM).

Supplementary Note S4

Calibration standards for multiplexed ion sensing in mixed solution

Applying a varying DC voltage to each inductor in the multiplexed sensing platform, performing linear fitting, and extracting the slope yield the voltage sensitivity (S_1, S_2, S_3) (unit: MHz/mV) and the following matrix (unit: mV/MHz) (Fig. 5C):

$$\mathbf{A} = \begin{bmatrix} \frac{1}{S_1} & 0 & 0 \\ 0 & \frac{1}{S_2} & 0 \\ 0 & 0 & \frac{1}{S_3} \end{bmatrix} = \begin{bmatrix} \frac{1}{0.03415} & 0 & 0 \\ 0 & \frac{1}{0.04926} & 0 \\ 0 & 0 & \frac{1}{0.06149} \end{bmatrix} (\text{mV/MHz}) \quad (S6)$$

Extracting the sensitivity of the three sensors to each ion based on results in Fig. 5G-I yields the following matrix:

$$\mathbf{S} = \begin{bmatrix} S(Na^{ISM}Na^{+}) & S(Na^{ISM}K^{+}) & S(Na^{ISM}H^{+}) \\ S(K^{ISM}Na^{+}) & S(K^{ISM}K^{+}) & S(K^{ISM}H^{+}) \\ S(H^{ISM}Na^{+}) & S(H^{ISM}K^{+}) & S(H^{ISM}H^{+}) \end{bmatrix}$$

$$= \begin{bmatrix} 2.05132 & 0.04412 & -0.30995 \\ 0.02199 & 2.88962 & 0.13283 \\ -0.02194 & 0.30857 & 2.52225 \end{bmatrix} (\text{MHz/dec}) \quad (S7)$$

The following equation converts unit of the sensitivity matrix from MHz/dec (**S**) to mV/dec (**S'**):

$$\mathbf{S'} = \begin{bmatrix} S'(Na^{ISM}Na^{+}) & S'(Na^{ISM}K^{+}) & S'(Na^{ISM}H^{+}) \\ S'(K^{ISM}Na^{+}) & S'(K^{ISM}K^{+}) & S'(K^{ISM}H^{+}) \\ S'(H^{ISM}Na^{+}) & S'(H^{ISM}K^{+}) & S'(H^{ISM}H^{+}) \end{bmatrix} = \mathbf{A} \times \mathbf{S}$$

$$= \begin{bmatrix} 60.06794 & 1.29195 & -9.07613 \\ 0.44641 & 58.66058 & 2.69651 \\ -0.35681 & 5.01824 & 41.01886 \end{bmatrix} (\text{mV/dec}) \quad (S8)$$

The following equation calculates the difference in surface potential of a test solution from the standard reference solution based on results in Fig. S21:

$$\begin{bmatrix} \Delta V(Na^{ISM}) \\ \Delta V(K^{ISM}) \\ \Delta V(H^{ISM}) \end{bmatrix} = \mathbf{A} \times \begin{bmatrix} \Delta f_s(Na^{ISM}) \\ \Delta f_s(K^{ISM}) \\ \Delta f_s(H^{ISM}) \end{bmatrix}$$

$$= \begin{bmatrix} \frac{1}{0.03415} & 0 & 0 \\ 0 & \frac{1}{0.04926} & 0 \\ 0 & 0 & \frac{1}{0.06149} \end{bmatrix}_{(\text{mV/MHz})} \times \begin{bmatrix} 0.3963 \\ 0.44914 \\ 0.522369 \end{bmatrix}_{(\text{MHz})}$$

$$= \begin{bmatrix} 11.604 \\ 9.118 \\ 8.495 \end{bmatrix}_{mV} \quad (S9)$$

The following equation calculates the concentration difference of a test solution from the standard reference solution based on results in Fig. S21:

$$\begin{bmatrix} \Delta[Na^{+}] \\ \Delta[K^{+}] \\ \Delta[H^{+}] \end{bmatrix} = \begin{bmatrix} S'(Na^{ISM}Na^{+}) & S'(Na^{ISM}K^{+}) & S'(Na^{ISM}H^{+}) \\ S'(K^{ISM}Na^{+}) & S'(K^{ISM}K^{+}) & S'(K^{ISM}H^{+}) \\ S'(H^{ISM}Na^{+}) & S'(H^{ISM}K^{+}) & S'(H^{ISM}H^{+}) \end{bmatrix}^{-1} \times \begin{bmatrix} \Delta V(Na^{ISM}) \\ \Delta V(K^{ISM}) \\ \Delta V(H^{ISM}) \end{bmatrix}$$

$$= \begin{bmatrix} 0.01668 & -0.00069 & 0.00373 \\ -0.00013 & 0.01715 & -0.00116 \\ 0.00016 & -0.00210 & 0.02455 \end{bmatrix}_{dec/mV} \times \begin{bmatrix} 11.604 \\ 9.118 \\ 8.495 \end{bmatrix}_{mV} = \begin{bmatrix} 0.219 \\ 0.145 \\ 0.191 \end{bmatrix} (dec)$$

(S10)

Alternatively, by combing Equation S6 – S10, the calibration can also be simplified as

$$\begin{bmatrix} \Delta[\mathrm{Na^+}] \\ \Delta[\mathrm{K^+}] \\ \Delta[\mathrm{H^+}] \end{bmatrix} = (\mathbf{A} \times \mathbf{S})^{-1} \times (\mathbf{A} \times \Delta\boldsymbol{f}_s) = \mathbf{S}^{-1} \times \mathbf{A}^{-1} \times \mathbf{A} \times \Delta\boldsymbol{f}_s = \mathbf{S}^{-1} \times \Delta\boldsymbol{f}_s = \begin{bmatrix} 0.219 \\ 0.145 \\ 0.191 \end{bmatrix} (dec)$$

(S11)

Table S3. Extracted values of f_s of the three sensors in Fig. 5D-F during cross-sensitivity tests in mixed ion solutions.

[H+] (μM)	fs_{Na+ISM} (MHz)	fs_{K+ISM} (MHz)	fs_{H+ISM} (MHz)
3.98	111.62	148.66	179.89
7.92	111.49	148.71	180.44
15.7	111.43	148.74	181.39

[K+] (mM)	fs_{Na+ISM} (MHz)	fs_{K+ISM} (MHz)	fs_{H+ISM} (MHz)
2.95	111.43	148.74	181.39
11.75	111.46	150.51	181.60
46.47	111.49	152.20	181.76

[Na+] (mM)	fs_{Na+ISM} (MHz)	fs_{K+ISM} (MHz)	fs_{H+ISM} (MHz)
4.84	111.49	152.20	181.76
19.31	112.78	152.17	181.82
76.34	113.94	152.23	181.74

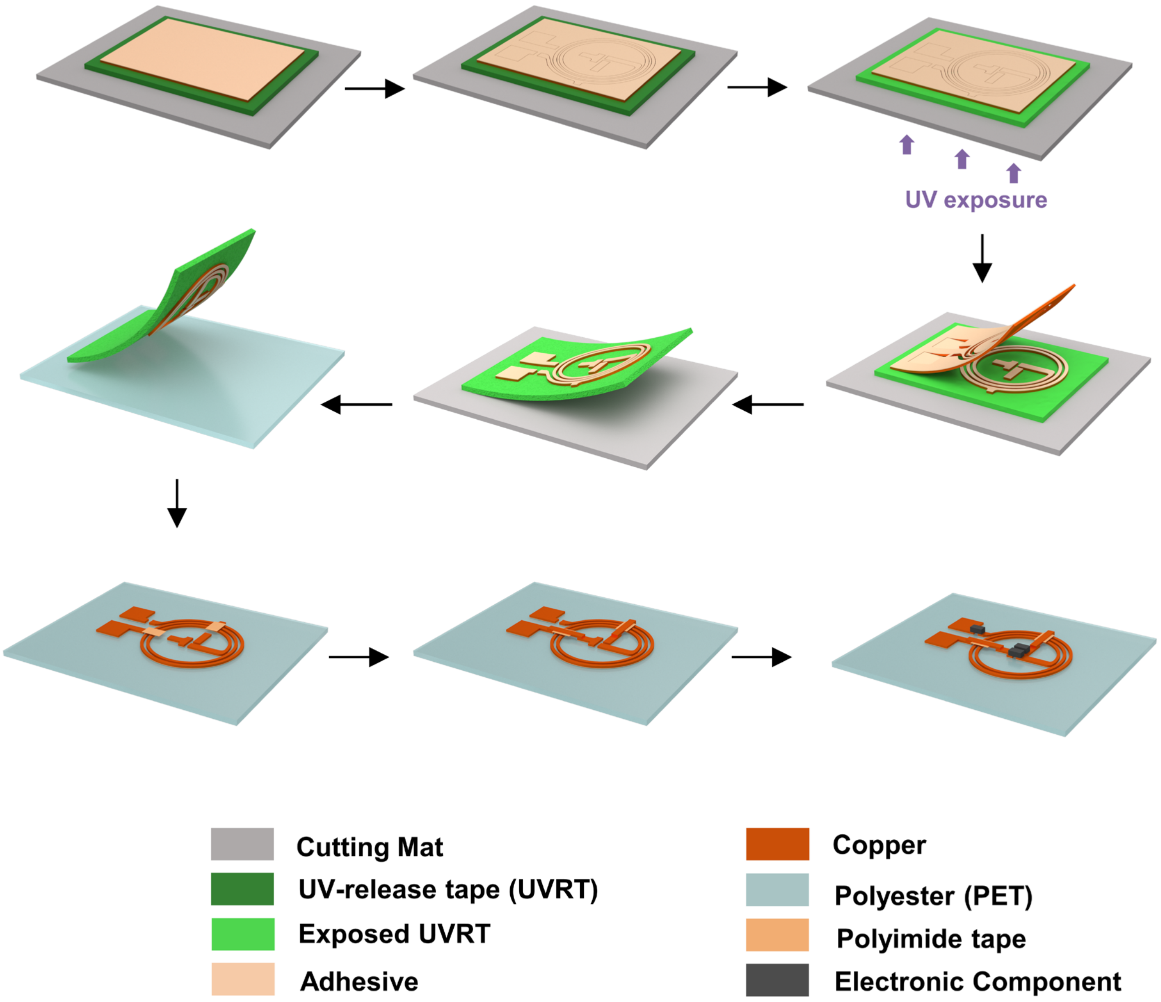

Fig. S1. Schematic illustration of fabricating circuit traces of the sensor system using the cut-and-paste method.

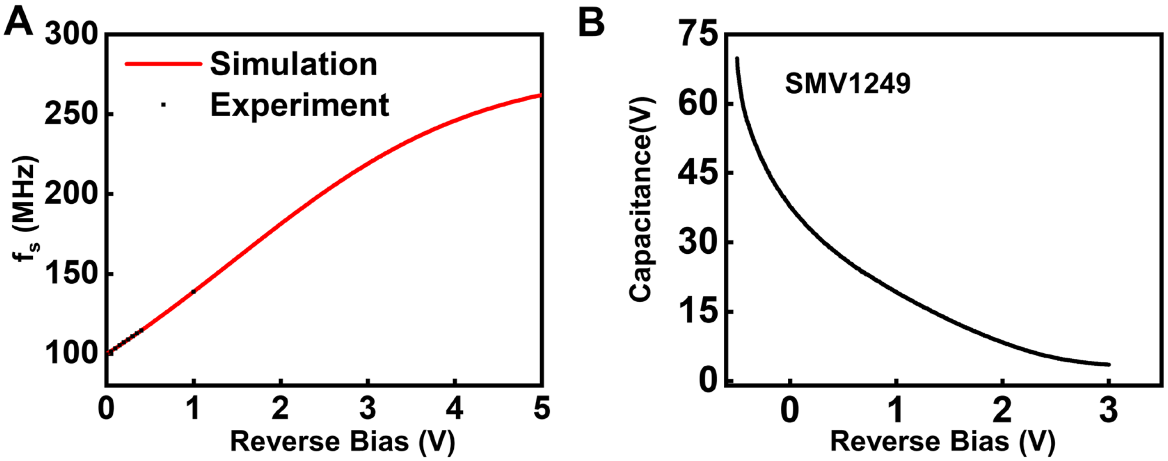

Fig. S2. Electrical characterization of the commercial hyper-abrupt varactor diode used in this study. (A) Simulated and measured results of f_s, and (B) measured capacitance of a hyper-abrupt varactor diode as a function of input reverse bias.

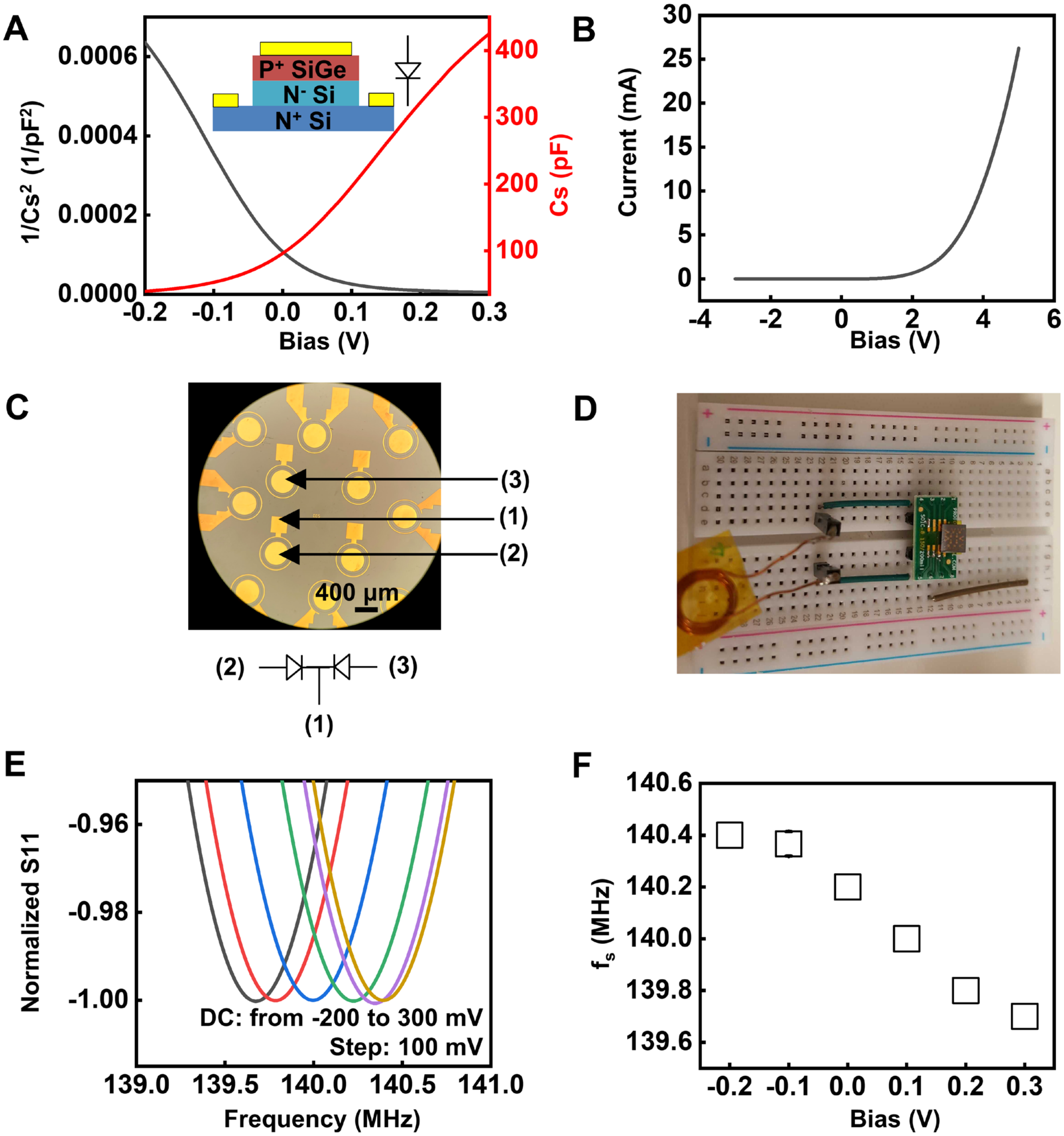

Fig. S3. Electrical characterization of the SiGe/Si diode. (A-B) *C-V* and *I-V* curves of the SiGe/Si diodes. The $1/C^2$-V plot shows a linear relationship under reverse bias. (C) Microscopic image of the diodes. (D) Layout of the LC circuit based on a pair of SiGe/Si diodes and a manually wrapped inductor coil. (E-F) Resonance curves and extracted f_s of the LC circuit with a DC voltage applied to the diodes.

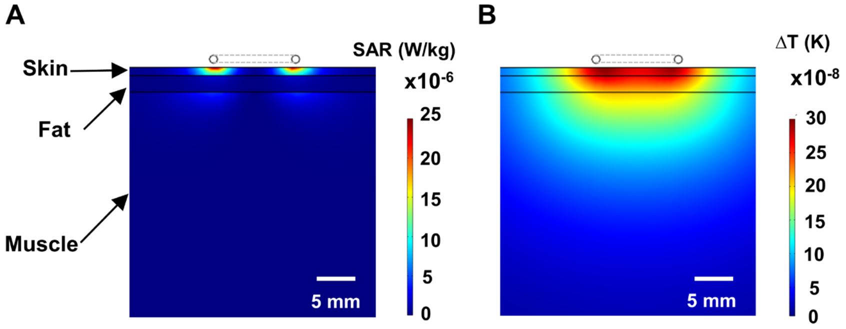

C

Medium	Thickness (mm)	Relative permittivity	Conductivity (S/m)	Thermal Conductivity (W/(m*K))	Density (kg/m^3)	Blood Perfusion rate (1/s)
Air	0.5	1	0	X	X	X
Skin	1	124	0.38	0.37	1109	0.0004
Fat	2	15.4	0.064	0.21	911	0.0005
Muscle	13	82.6	0.67	0.49	1090	0.00042

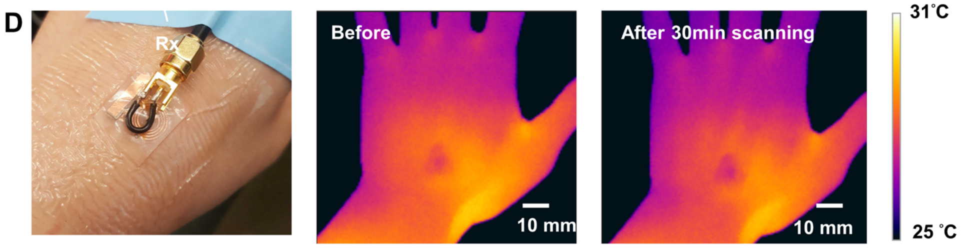

Fig. S4. Electromagnetic and thermal safety of the wireless system. (A-B) Simulated SAR and heat generation with an RF power of -9 dBm transmitted from the readout coil. The two dots on the top represent the single turn coil antenna. (C) Parameters used for the air and different tissue layers (from top to bottom) in the simulations. (D) Photograph and infrared (IR) thermal camera images of a sensor deployed on human skin before and after continuous operation for 30 min. The experiment includes a sensor device laminated on the skin and radiated by a transmitter coil connected to a VNA (diameter = 10 mm, -9 dBm, 100-120 MHz). The distance between the transmitter coil and skin is 0.5 mm. An IR camera (FLIR i7) captures the images before and after 30 min of radiation.

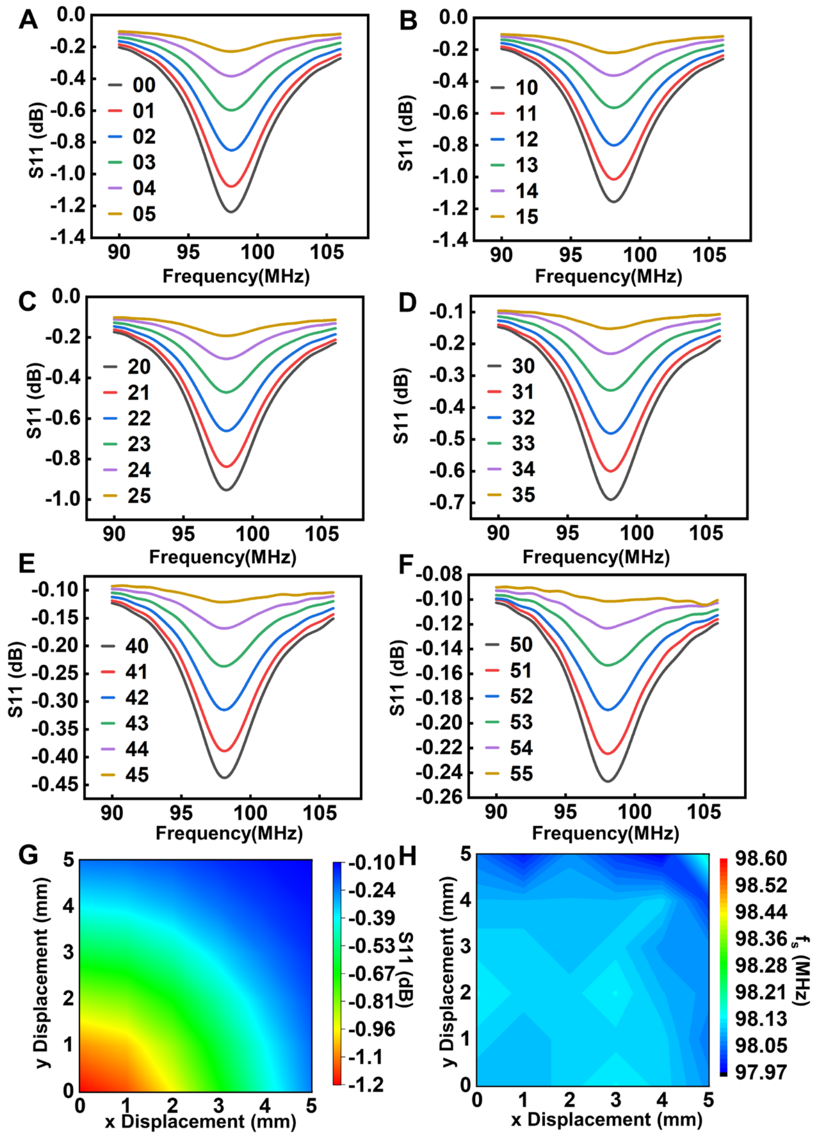

Fig. S5. Characterization of the working range of the sensor in Fig. 2. (A-F) Resonance curves upon systematically varying the relative position between the antenna coupler and the readout coil. (G-H) Mapping of *S11* (dB) (G) and f_s (H) as a function of the lateral distance from the origin (z = 5 mm).

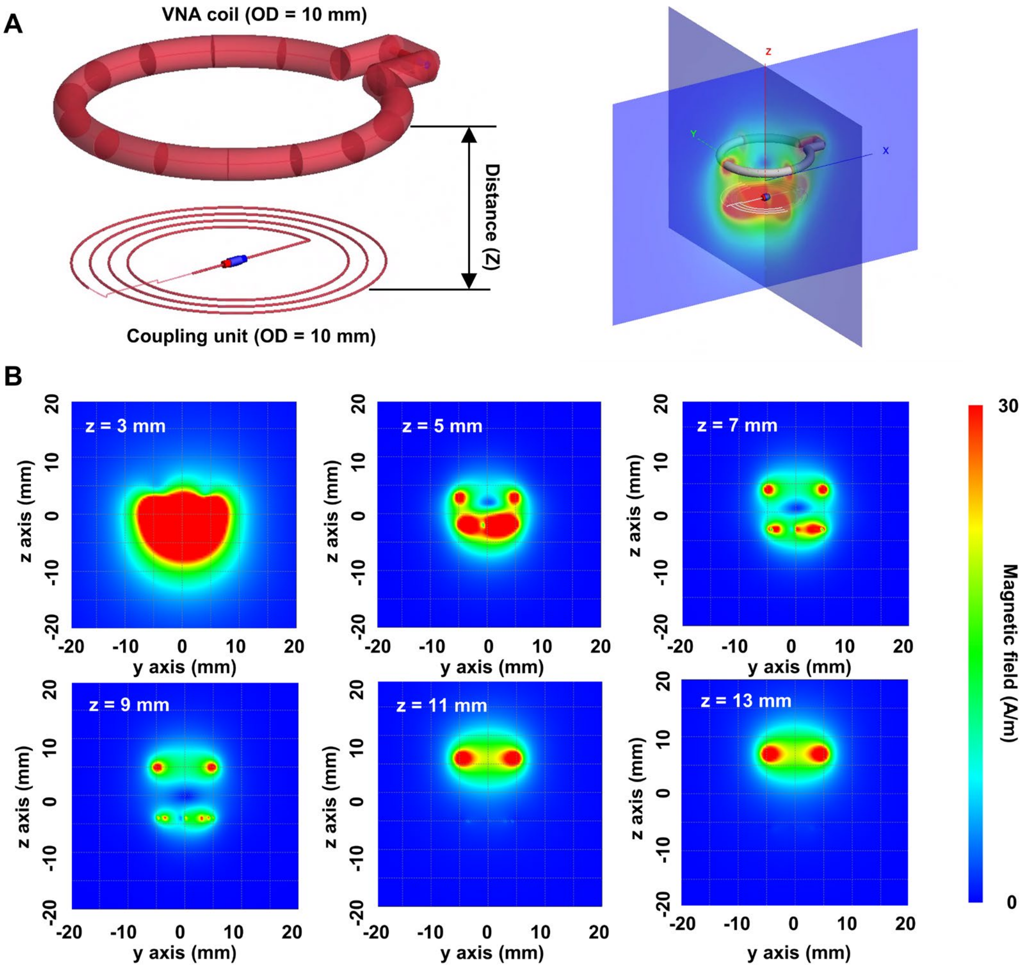

Fig. S6. Results of near-field simulation showing the strength of the magnetic field around the inductor coil. The LC oscillator tank consists of a three-turn inductor and a 20 pF capacitor.

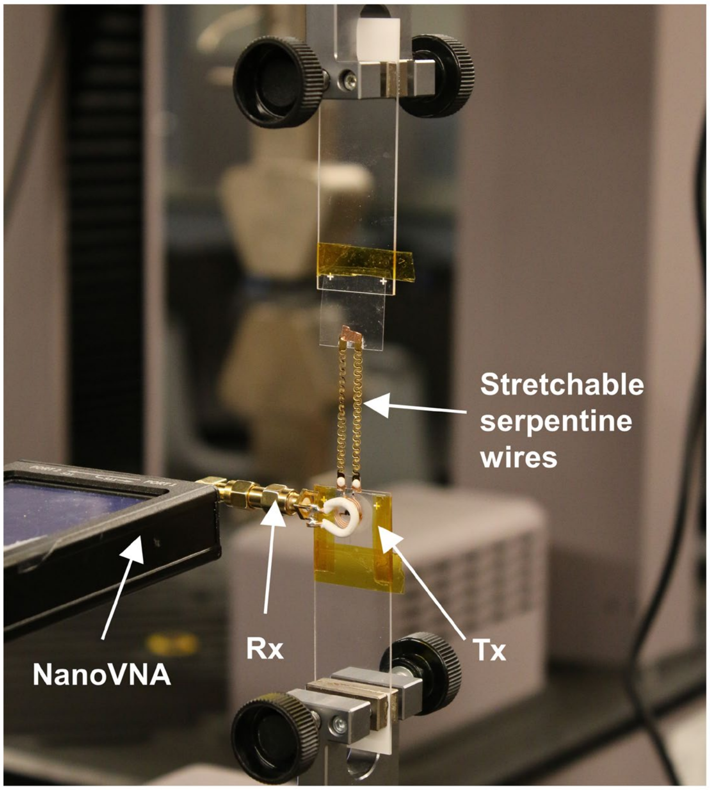

Fig. S7. Photograph showing the experimental setup for mechanical testing using a tensile tester.

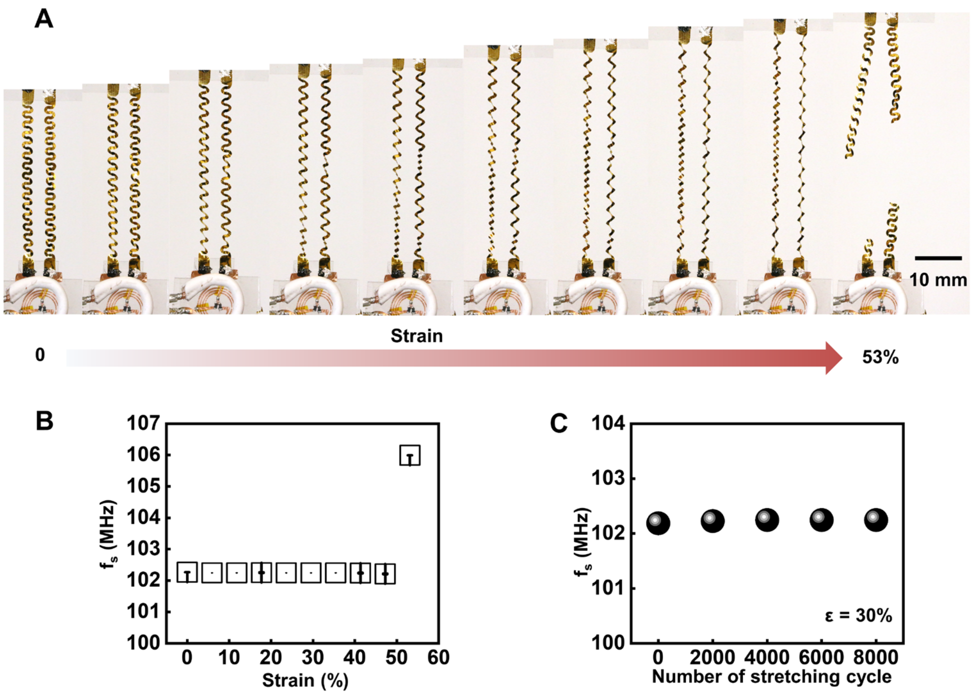

Fig. S8. Extended data showing the mechanical robustness and reliability of the sensor system. (A-B) Photographs and extracted f_s of a test device in the presence of a strain up to 50%. The DC part of the device fractures at a strain of ~53%. (C) f_s of the device sensor before and after 0-8000 stretch cycles with an applied tensile strain of 30%

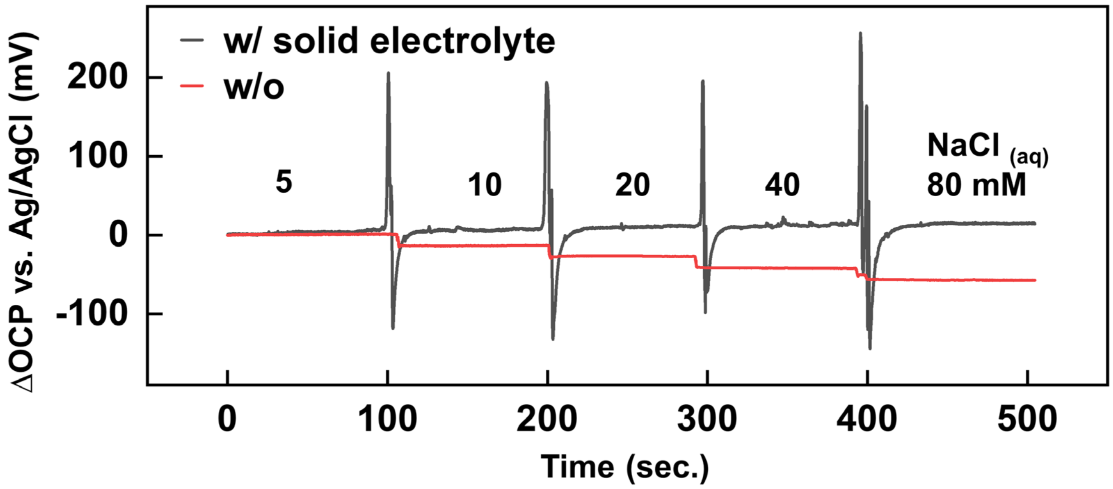

Fig. S9. Stability test results in solution of thin-film Ag/AgCl reference electrode with and without encapsulation of solid electrolytes. Varying the concentration of NaCl (5-80 mM) in the solution examines the stability of the two types of reference electrode, suggesting the importance to have the encapsulation layer of solid electrolytes.

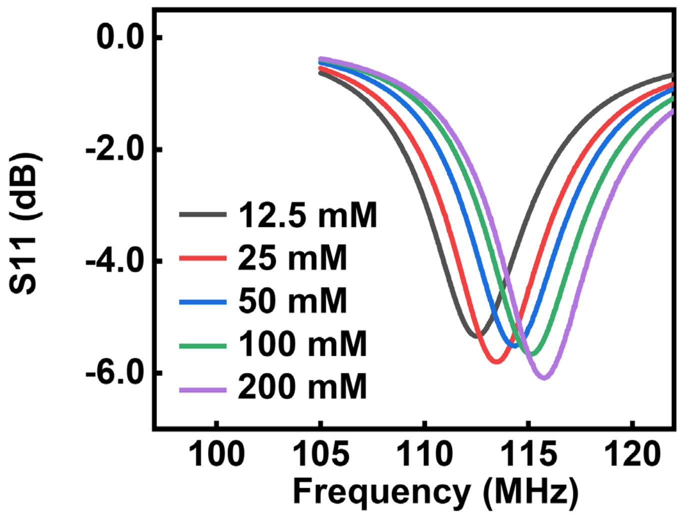

Fig. S10. Raw data of measured *S11* of the wireless K^+ sensor in Fig. 3D (before normalization).

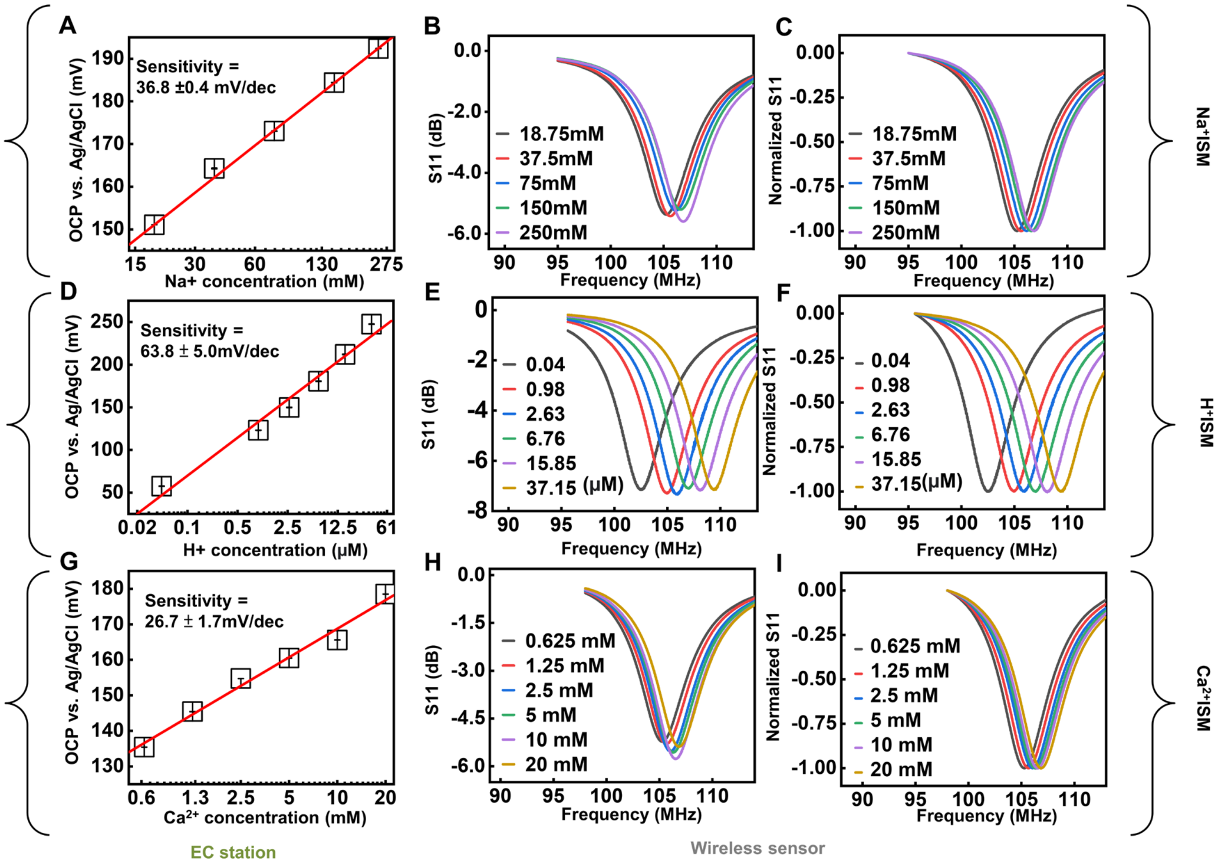

Fig. S11. Sensing performance of Na^+ (A-C), H^+ (D-F), and Ca^{2+} (G-I) -ISM functionalized sensors in response to solutions with corresponding target ions.

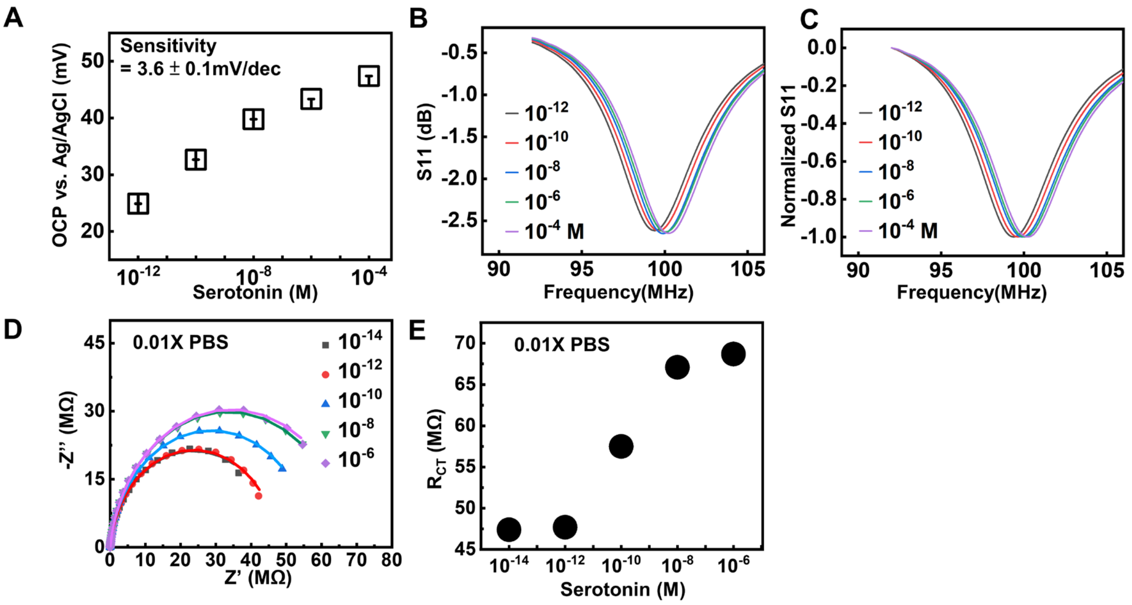

Fig. S12. Sensing performance of anti-serotonin aptamer functionalized sensors in response to serotonin in 0.01X PBS. (A) OCP (vs. Ag/AgCl reference electrode) of the sensing interface as a function of serotonin concentration in 0.01X PBS solution. (B-C) Resonance curves of a wireless sensor system integrated with an anti-serotonin sensing interface in response to serotonin in 0.01X PBS solutions (before and after normalization). (D) EIS characterization of an aptamer-functionalized electrode in 0.01X PBS solutions with varying concentration of serotonin. (E) Charge transfer resistance extracted from EIS results in Fig. S12D.

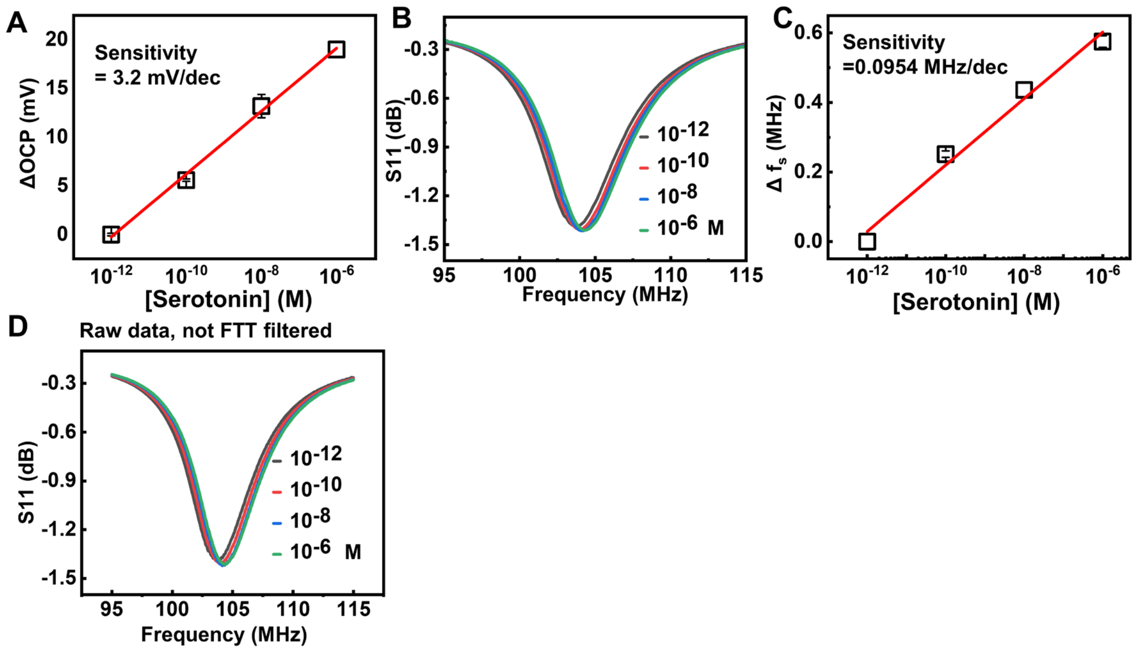

Fig. S13. Sensing performance of anti-serotonin aptamer functionalized sensors in 0.3× PBS solution (ionic strength: 51.45 mM). (A) OCP (vs. Ag/AgCl reference electrode) of the sensing interface as a function of serotonin concentration in 0.3× PBS solution. (B-C) Resonance curves and extracted f_s of a wireless sensor system integrated with an anti-serotonin sensing interface in response to serotonin in 0.3× PBS solutions. (D) Raw data of the resonance curves before FFT filtering.

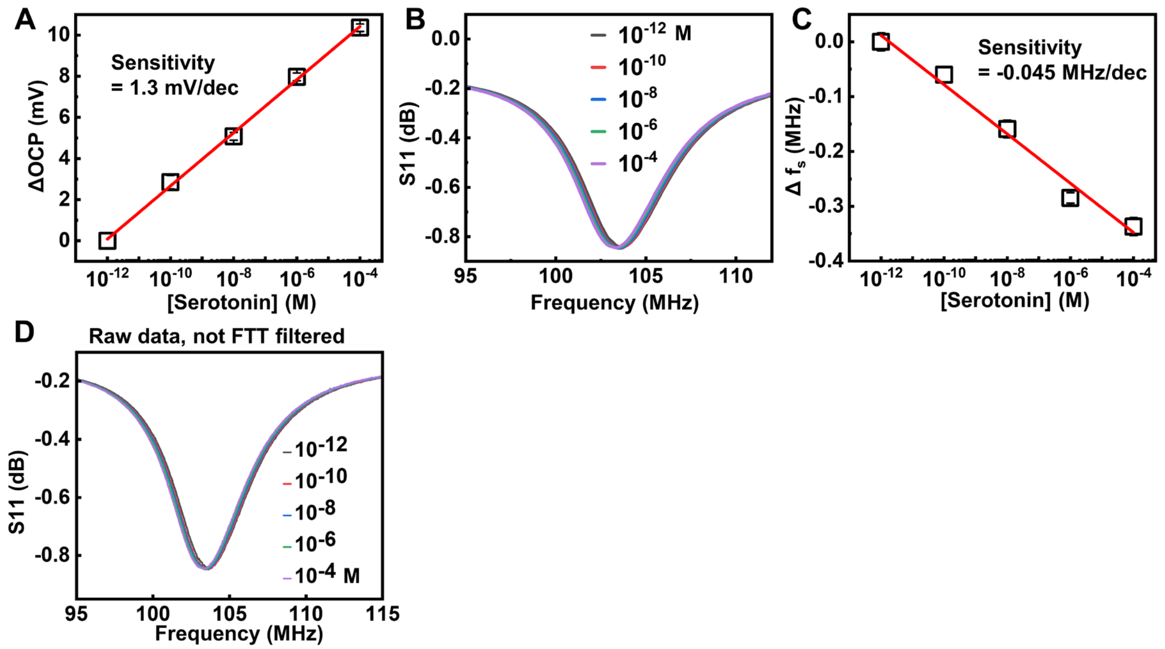

Fig. S14. Sensing performance of anti-serotonin aptamer functionalized sensors in 1X PBS solution in the presence of dopamine (1 ng/mL) and glutamate (1 µM). (A) OCP (vs. Ag/AgCl reference electrode) of the sensing interface as a function of serotonin concentration. (B-C) Resonance curves and extracted f_s of a wireless sensor system integrated with an anti-serotonin sensing interface in response to serotonin. Please note that different from other serotonin sensors reported in this study, in the presence of dopamine (1 ng/mL) and glutamate (1 µM), the OCP value remains negative (instead of positive) throughout the dynamic range. Therefore, the sensor has the anode and cathode of the varactors connected to SE and RE, respectively, such that the voltage serves as the reverse bias for the varactors. The leads to a negative value of the sensitivity (-0.045 MHz/dec). (D) Raw data of the resonance curves before FFT filtering.

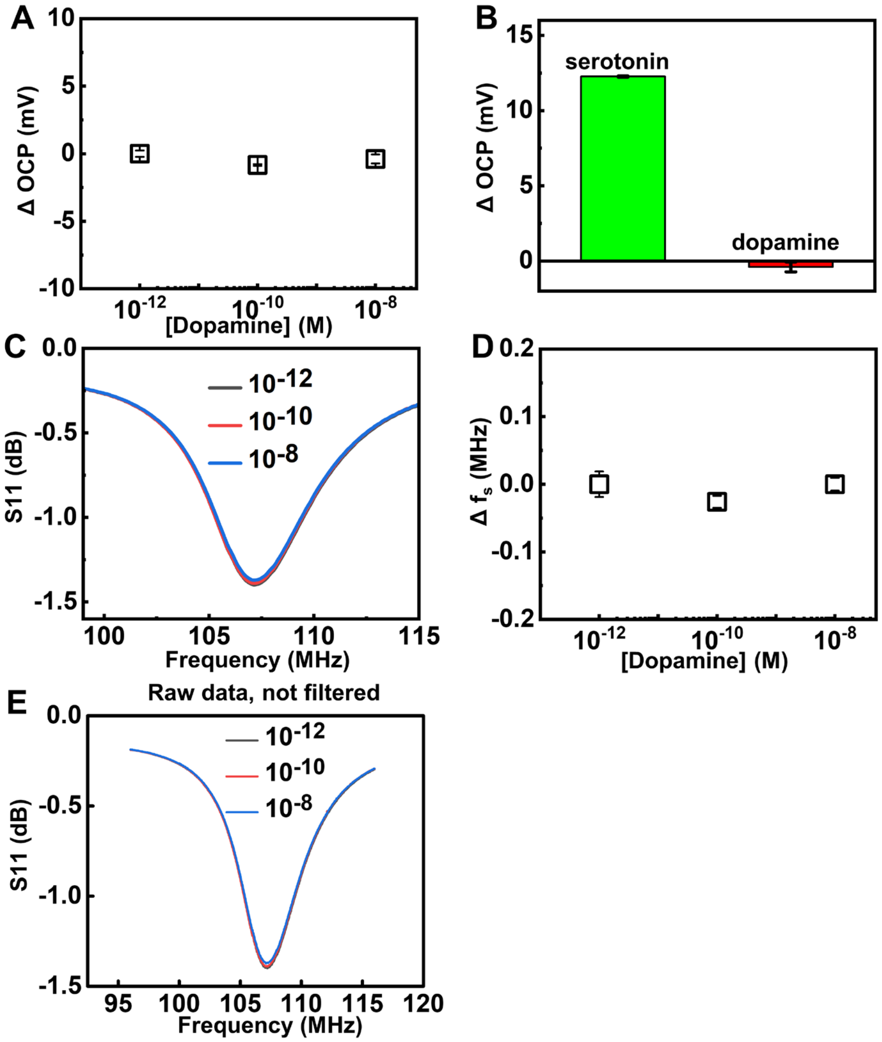

Fig. S15. Sensing performance of anti-serotonin aptamer functionalized sensors in 1X PBS solution with dopamine (concentration: 10^{-12} to 10^{-8} M). (A) OCP (vs. Ag/AgCl reference electrode) of the sensing interface as a function of dopamine concentration. (B) Comparison of the response of the anti-serotonin sensor to serotonin and dopamine within the same concentration range (10^{-12} to 10^{-8} M). (C-D) Resonance curves and extracted f_s of a wireless sensor system integrated with an anti-serotonin sensing interface in response to dopamine. (E) Raw data of resonance curves before FFT filtering.

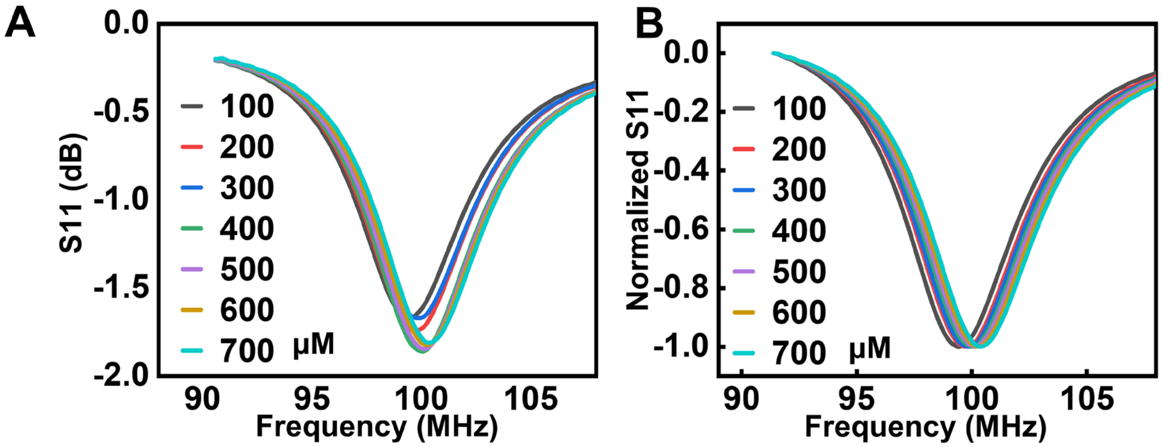

Fig. S16. Extended data for sensing performance of the glucose oxidase functionalized wireless sensor in Fig. 4I in response to glucose solution (before and after normalization).

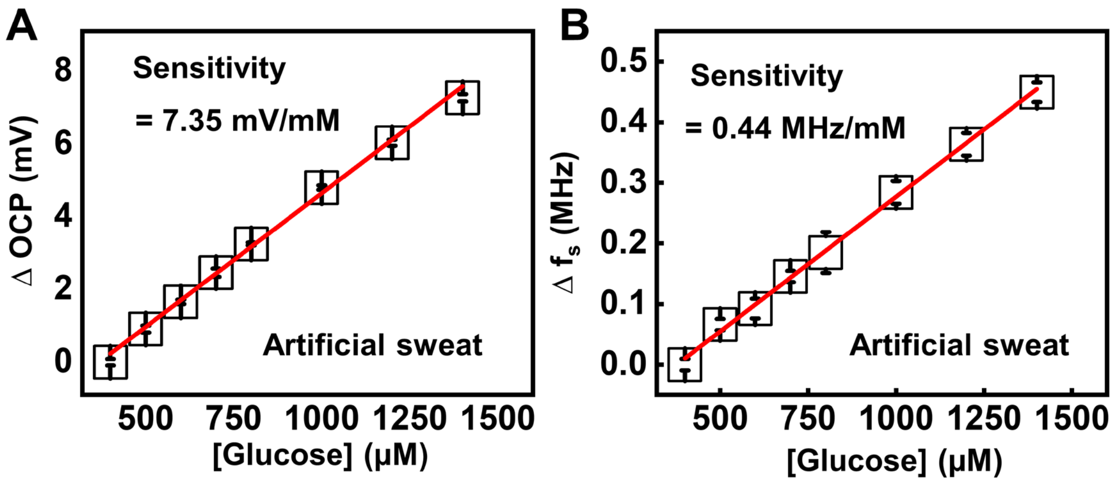

Fig. S17. Sensing performance of a GOx functionalized sensor in commercial artificial sweat. Signal readout in terms of OCP (A) and f_s (B) as a function of glucose added into the system.

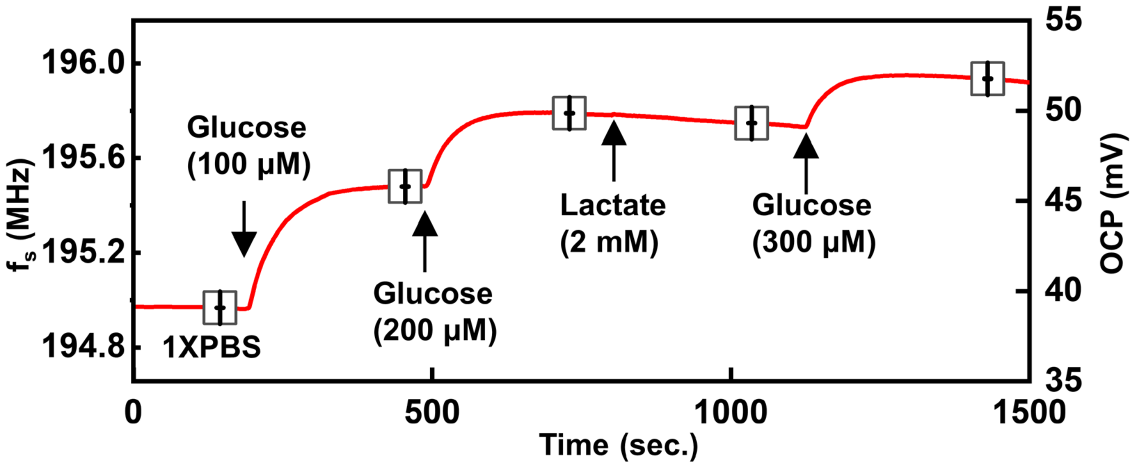

Fig. S18. Realtime signal readout (f_s and OCP) of a GOx functionalized sensor in response to glucose and lactate. The results suggest a minimal response of the sensor to the addition of lactate (2 mM) added into the system, indicating its high specificity to glucose.

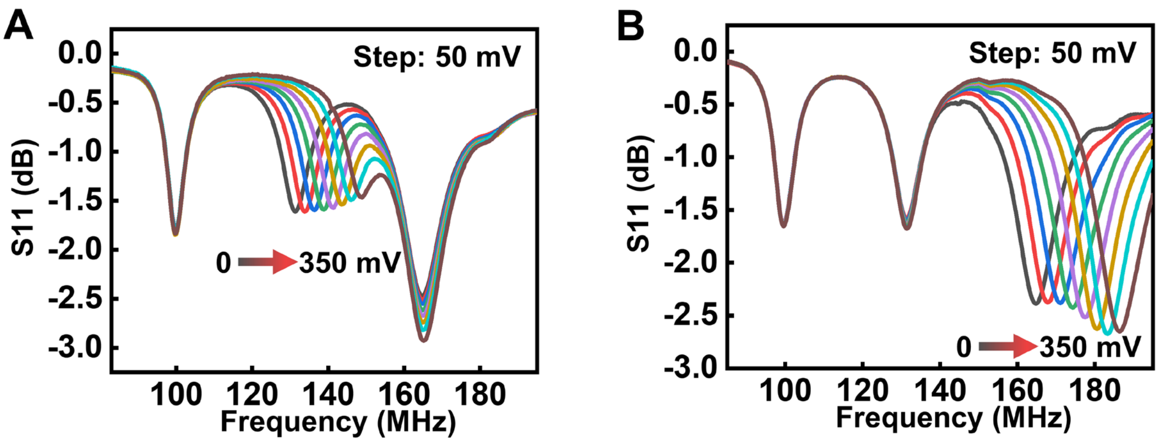

Fig. S19. Resonance curves of sensor 2 and sensor 3 in the multiplexed sensing platform in Fig. 5A showing the response to an input DC bias.

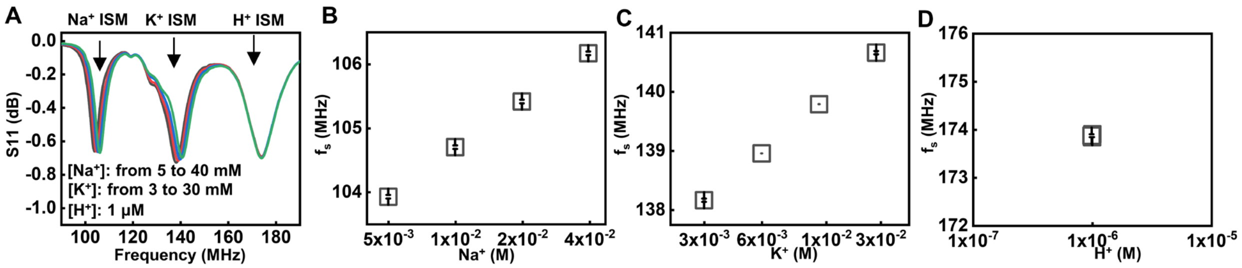

Fig. S20. Experimental results obtained using a three-sensor system with the concentration of Na^+ and K^+ changing at the same time. (A) Resonance curves of the three-sensor system before and after the concentration change. (B-D) Summary of changes in f_s of the three sensors.

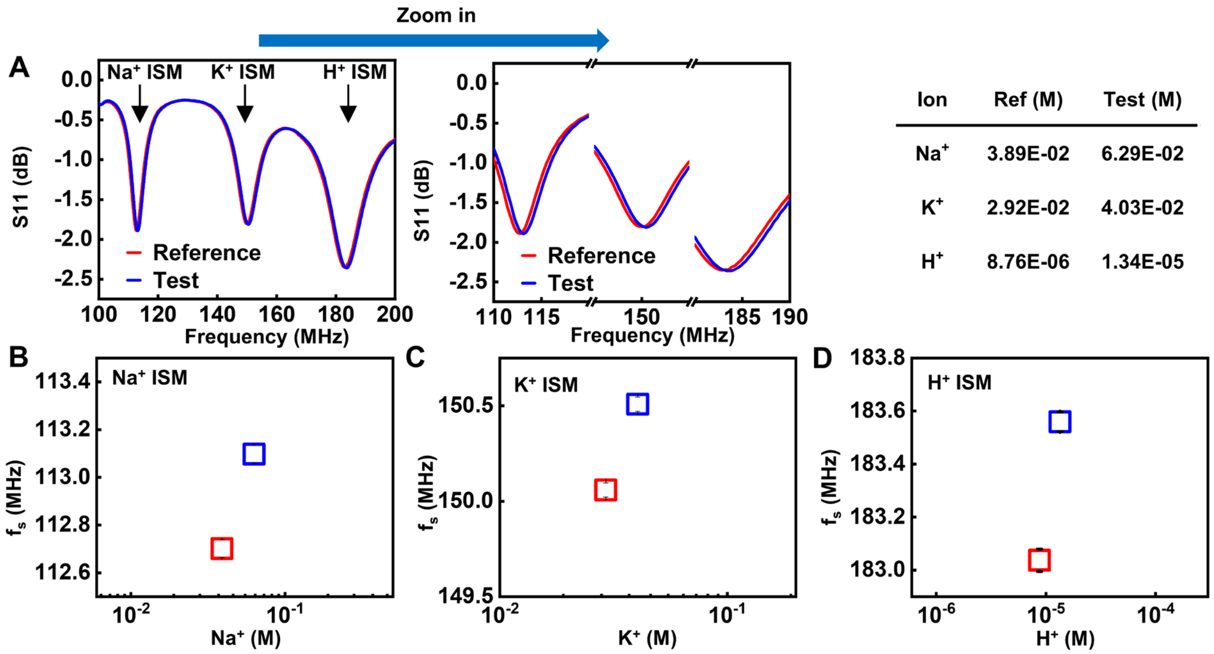

Fig. S21. Calibration of sensing results based on the sensitivity matrix and readouts of the multiplexed sensor array in reference and test solutions. (A) Resonance curves of the multiplexed sensor array in reference and test solutions, and actual concentration of each ion in the reference and test solutions. (B-D) Extracted f_S of the three sensors in the reference and test solutions. $\Delta \boldsymbol{f}_S$ is then used for calibration according to Supplementary Note S3 in combination with the sensitivity matrix $\boldsymbol{S}$. The ion concentrations in the reference solution are used to calculate the measured ion concentrations in the test solution based on $\Delta \boldsymbol{C}$. The actual concentrations of the test solution are used to calculate the errors.

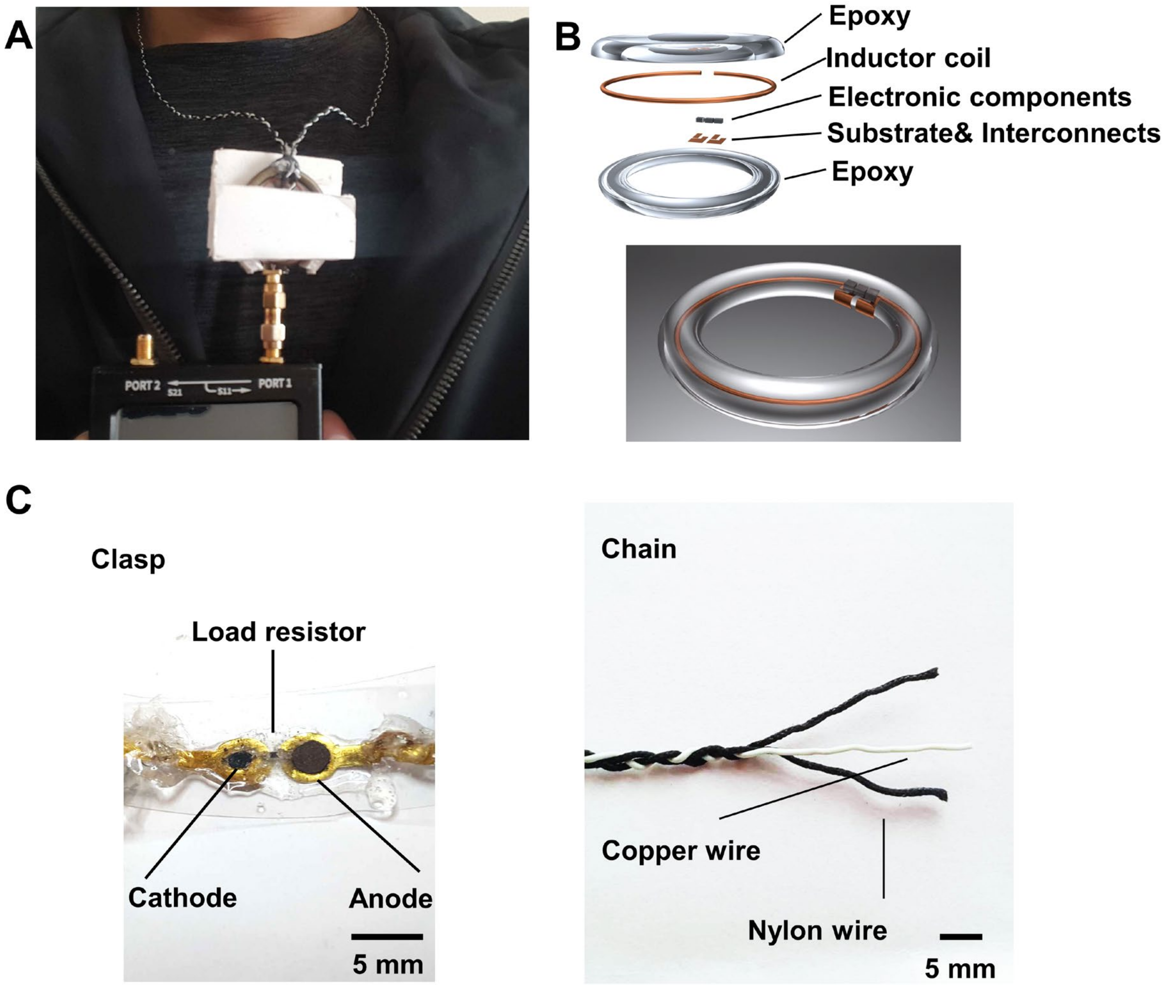

Fig. S22. Schematic illustration and photographs of the "smart necklace" and measurement setup with a portable NanoVNA. (A) A subject reading the signal during exercise using a portable VNA. To improve the stability of the system, a self-made spacer keeps the distance between the "pendant" and readout coil constant. (B) Exploded view schematic illustration showing the design and key functional layers of the "pendant". (C) Photographs of the "clasp" and "chain".
